# Understanding salinity responses and adopting ‘omics-based’ approaches to generate salinity tolerant cultivars of rice

**DOI:** 10.3389/fpls.2015.00712

**Published:** 2015-09-09

**Authors:** Priyanka Das, Kamlesh K. Nutan, Sneh L. Singla-Pareek, Ashwani Pareek

**Affiliations:** ^1^Stress Physiology and Molecular Biology Laboratory, School of Life Sciences, Jawaharlal Nehru UniversityNew Delhi, India; ^2^Plant Molecular Biology Group, International Centre for Genetic Engineering and BiotechnologyNew Delhi, India

**Keywords:** genomics, *Oryza sativa*, proteomics, salinity, transcriptomics, yield

## Abstract

Soil salinity is one of the main constraints affecting production of rice worldwide, by reducing growth, pollen viability as well as yield of the plant. Therefore, detailed understanding of the response of rice towards soil salinity at the physiological and molecular level is a prerequisite for its effective management. Various approaches have been adopted by molecular biologists or breeders to understand the mechanism for salinity tolerance in plants and to develop salt tolerant rice cultivars. Genome wide analysis using ‘omics-based’ tools followed by identification and functional validation of individual genes is becoming one of the popular approaches to tackle this task. On the other hand, mutation breeding and insertional mutagenesis has also been exploited to obtain salinity tolerant crop plants. This review looks into various responses at cellular and whole plant level generated in rice plants toward salinity stress thus, evaluating the suitability of intervention of functional genomics to raise stress tolerant plants. We have tried to highlight the usefulness of the contemporary ‘omics-based’ approaches such as genomics, proteomics, transcriptomics and phenomics towards dissecting out the salinity tolerance trait in rice. In addition, we have highlighted the importance of integration of various ‘omics’ approaches to develop an understanding of the machinery involved in salinity response in rice and to move forward to develop salt tolerant cultivars of rice.

## Introduction

Today’s agriculture faces a daunting task of ensuring food security to the increasing human population on this planet (FAO, 2009). A great proportion (more than 60%) of this population depends on rice (*Oryza sativa* L.) as their staple food. Rice contributes up to 20% of the calories consumed by human nutrition worldwide. Therefore, rice production must increase during the coming time in order to keep pace with increasing world population. Asia is known as the main rice producer in the world by yielding more than 650 million tons (90% of total rice yield worldwide) grown in 145 million ha land.

Rice is grown in a diverse range of environments characterized by various climates and soil-water conditions. However, adverse environmental conditions critically threaten rice production and causes significant yield loss in large areas of main productive sectors. Both abiotic and biotic stresses frequently prevent the attainment of optimum growth and yield of rice. These stresses include high salinity, drought, heat, and cold which have negative effect on the yield and vegetative production of rice, and cause a key risk to worldwide food safety (Pareek et al., 2010; Mantri et al., 2012).

Amongst the various environmental stress factors, salinity is the main hazardous factor limiting crop productivity. Rice has been grouped as salinity susceptible cereal at its young stage (Lutts et al., 1995) and confines its efficiency of production at mature stage (Todaka et al., 2012). To increase the grain yield of rice under salinity, it is imperative to first understand the basic molecular machineries of salt tolerance in this plant. Tolerance toward salinity is a quantitative attribute in plants, regulated by a host of genes (Chinnusamy et al., 2005). Since the last decade, numerous genes imparting salinity tolerance in plants (including rice) have been identified and characterized such as those involved in transcription regulation, signal transduction, ion transportation and metabolic homeostasis (Verma et al., 2007; Singh et al., 2008; Singla-Pareek et al., 2008; Kumari et al., 2009). In the present text, we present our current understanding about effects of soil salinity on rice crop and the approaches used to increase the tolerance of this crop toward salinity. Further, critical evaluation of progress made toward raising salinity tolerant rice using functional genomics tools is also presented.

## Soil Salinity as an Obstacle in Plant Growth, Photosynthesis, and Grain Yield

Salinization is one of the severe soil degradation factors. Approximately 6.5% of world’s total area and about 20 percent of the cultivated area is already affected by soil salinity (Hakim et al., 2014). Saline area is increasing due to various factors including natural reasons as well as human activities. As per Reynolds et al. (2001), accretion of salts in the soil surface is caused by different factors in different geological and climatic regions. Salinity is frequently accompanied by water logging and alkalinity, which apply their individual specific effects on plant development (Yeo, 1999). Crop plants show a spectrum of reactions toward salinity including reduced growth and yield. Plants responses toward salinity is the collective outcome of the intricate communications among various processes linked to plant morphology, biochemistry, and physiology.

In most of the plants, obvious signs of damage by salinity are growth inhibition, senescence and death through long-standing exposure. Inhibition in seedling/plant growth is the initial step that leads to other indications, even though, programmed cell death may also take place under severe salinity. Salinity induces abscisic acid synthesis which leads to stomatal closure, reduced photosynthesis and photoinhibition. An instant outcome of salinity on plant development is inhibition of cell growth through abscisic acid synthesis.

Overloaded sodium ions around the root exterior disturb uptake of potassium. Because of the identical chemical properties of Na^+^ and K^+^, Na^+^ has a negative effect on K^+^ uptake. Under Na^+^ stress, it is essential for plants to activate and maintain high-affinity K^+^ uptake machinary rather than low affinity K^+^ uptake one in order to uphold sufficient K^+^ concentration in the cell. Shortage of potassium inside the cell unavoidably leads to decrease in plant growth, as K^+^ is the most abundant cellular cation which plays an important role in preserving membrane potential, enzyme activities and cell turgor (Xiong and Zhu, 2002). After entering the cytosol, Na^+^ inhibits the activity of an array of enzymes/proteins (Xiong and Zhu, 2002). This inhibition is K^+^ dependent in the cell: a high Na^+^/K^+^ ratio cause damage to the cell. Accretion of Na^+^ in the apoplast slowly increases the osmotic gradient connecting the out- and in-side of the cell. To attain balance, water from inside of the cell moves outward into the intracellular spaces which cause cellular dehydration and ultimately, cell death. Constant contact of root with high salinity gradually decreases leaf size (Munns and Tester, 2008).

The effect of soil salinity upon photosynthesis process at its vegetative as well as reproductive stage has been studied by many researchers (Yeo et al., 1985; Dionisio-Sese and Tobita, 2000; Senguttuvel et al., 2014). It has been established that photosynthesis and chlorophyll concentration are inversely correlated with level of salt stress (Senguttuvel et al., 2014). Furthermore, it has also been reported that particular concentration of sodium chloride in the leaf causes reduction in photosynthesis to its half without affecting the concentration of chlorophyll (Dionisio-Sese and Tobita, 2000). Tolerance of crops to abiotic stresses depends upon their chlorophyll stability index. Salinity did not have much effect on the chlorophyll contents of the tolerant cultivars because they contain high chlorophyll stability index (Mohan et al., 2000; Sikuku et al., 2010). Similarly, ratio of chlorophyll-a and -b in plants also decrease due to salt stress (Senguttuvel et al., 2014). Unlike salinity susceptible varieties, tolerant varieties always maintain chlorophyll a/b ratio under salt stress conditions. Chlorophyll fluorescence parameters have also been found to disturbed due to salinity. It has been observed that the tolerant cultivars maintain a high Fv/Fm ratio than the susceptible one (Senguttuvel et al., 2014).

In plants, the harvest index (amount of shoot mass and yield) can fluctuate from 0.2 to 0.5, depending on the harshness of salinity (Husain et al., 2003). A small concentration of salt do not decrease plant’s reproductive yield (although the plant’s vegetative biomass is decreased) which is revealed in harvest index that goes up with salt stress. It has been established that grain yield in many crops do not reduce until a threshold salinity level is reached (‘bent stick’ relationship; USDA-ARS, 2005). A survey in USA (USDA-ARS, 2005) has shown that the yield of rice starts to decrease at 30 mM NaCl whereas in wheat, 60–80 mM NaCl could result in decline of the grain yield. This study shows the genetic difference among species. For instance, huge genetic dissimilarity has been found in durum wheat and barley, developed by irrigation with altered salt levels (up to 250 mM; Royo and Abio, 2003). These experiments show a sigmoidal curve instead of a ‘bent stick’ association between the level of salinity and the crop yield. It has been reported that salinity reduces the efficiency of yield by reducing the formation of tillers (Maas and Hoffman, 1977). For various soils, salinity and waterlogging are interlinked. In Pakistan, use of high salt containing irrigation water causes poor soil texture and poor permeation of water (Qureshi and Barrett-Lennard, 1998). Secondary salinity occurs in Australia, where water-table increases to two meters of the soil layer which is near to the root sector. Moreover, as the porosity of soil is around 10 percent, it needs a little (∼100 mm) rainfall for water-table to increase up to the exterior surface to cause salinity and waterlogging stresses simultaneously (Barrett-Lennard, 2002).

## Salinity Response is Highly Complex and Determined by Developmental Stages of the Rice Plant

Salinity is one of the key obstructions of rice production worldwide. Rice is especially grouped under salt-sensitive crop (Shannon et al., 1998). There are two important factors (threshold and slope) enough for determining salinity tolerance. Threshold indicates highest permissible salt without reduction in yield and slope indicates percent of reduction in yield per unit rise in salt level ahead of the threshold. The threshold of rice is 3.0 dsm^-1^ and slope is 12% per dsm^-1^ (Maas and Hoffman, 1977). In addition, rice is also differentially affected by salt stress at various growth stages. Moreover, the adverse effect of salinity on development of rice plant has been found to be related to different growth stages of the plant, type of salt, concentration of salt, exposure period of salt, water regime, pH of soil, humidity, solar radiation and temperature (Akbar, 1986). It has been established that rice plant is comparatively tolerant to salt stress during seedling stage, as at this stage, the injury can be considerably overcome in the later phases of development (Akbar and Yabuno, 1974). Hence, seedling stage is the ideal stage to categorize the rice genotypes into various groups based on their tolerance toward salinity. The rice genotypes have been categorized into different groups from extremely tolerant (score 1) to extremely sensitive (score 9) (**Table 1**). Janaguiraman et al. (2003) have shown that salinity tolerant rice varieties have higher rate of germination, shoot length, root length, and vigor index.

**Table 1:** Phenotypic trends of various rice genotypes under control and salinity treatment (Source; Mohammadi-Nejad et al., 2010).

Rice variety	Score	Pollen viability		Grain yield (g)	
		Control	Salinity	Control	Salinity
IR65209-3B-6-3-1	1	46.7	14.6	1.4	1.0
IR65858-4B-11-1-2	1	48.1	48.0	4.4	0.8
IR69588-4R-P-11-3	1	36.6	18.8	2.0	0.5
IR72046-B-R-7-3-1-2	1	49.4	24.7	1.3	0.1
IR71832-3R-2-2-1	1	19.5	12.3	1.7	0.4
IR71899-2-1-1	1	34	14.9	2.6	1.1
IR71991-3R-2-6-1	1	27.8	22.1	3.6	1.4
IR71995-3R-1-2-2	1	38.2	15.7	3.8	2.6
IR74099-3R-3-3	1	52.3	19.7	1.9	0.8
IR74105-3R-2-1	1	54.9	19.5	0.6	0.2
IR70023-4B-R-12-3-1	1	81.2	46.2	2.7	1.9
Cheriviruppu	3	59.4	20.8	6.8	4.2
Kala Rata 1-24	1	69	48.6	3.1	1.5
Bhirpala	5	45.1	26.2	2.1	1.4
IR4630-22-2-5-1-3	5	60.1	45.6	1	0.7
Pokkali (Ac.108921)	1	67.7	29.6	5.5	5.2
IR66946-3R-178-1-1	1	73	47.4	2.7	0.5
IR64	3	46.6	8.2	0.9	0.9
IR65185-3B-8-3-2	5	16	19.7	2.7	0.6
IR72046-B-R-4-3-2-1-2B-1	3	48.8	18.2	2.2	0.1
IR72043-B-R-6-3-3-3	5	39.3	15.4	1.5	0.3
IR72046-B-R-8-3-1-3	5	47.4	17.2	3.8	0.8
IR75000-69-2-1	5	50.6	26.4	1.8	1.6
IR29	9	24.7	18.3	0.2	0.2
Mojang Kor	7	57.9	27.6	1	0.5
Bao thai	9	24.9	13.8	0.3	0.3
CN499-160-13-6	9	42.2	26.9	3.9	2.0
Karuna	9	36	33.4	0.6	0.2
TCA4	7	29.8	16.5	2.1	0.6
Kinandang Patong	7	56.4	27.4	1.2	0.5

Besides the seedling stage, flowering stage is another highly sensitive growth stage in the life cycle of crop plants which is affected by salinity stress (Singh et al., 2004). This stage is vital as it determines grain yield. Salinity stress at booting stage affects the pollen viability which results in poor fertilization and consequent reduction in the percentage of filled grains and hence, the total plant yield. In a recent study targeting to access the effect of salinity on the pollen viability and grain yield in rice using various genotypes of rice (**Table 1**) (Mohammadi-Nejad et al., 2010). It has been found that most of the rice varieties show reduced pollen viability under salinity but those which have severe reduction in the pollen viability, along with severe decrease in plant yield, were classified as the salinity-susceptible genotypes for flowering stage (Khatun and Flowers, 1995). Some landraces such as Kalarata, Cheriviruppu, Pokkali, and Bhirpala have been found to be comparatively tolerant at flowering stage due to better viability of pollens and higher grain yield (upto 49%) under salt stress. Similarly, some of the other rice varieties i.e., IR72046-B-R-7-3-1-2, IR4630-22-2-5-1-3, and CN499-160-13-6 have also been categorized as salt tolerant at flowering stage based on their pollen viability and grain yield. Two rice genotypes (IR66946-3R-178-1-1 and IR65858-4B-11-1-2) which show high pollen viability and less grain yield have been categorized as sensitive for the flowering stage.

**FIGURE 1:**
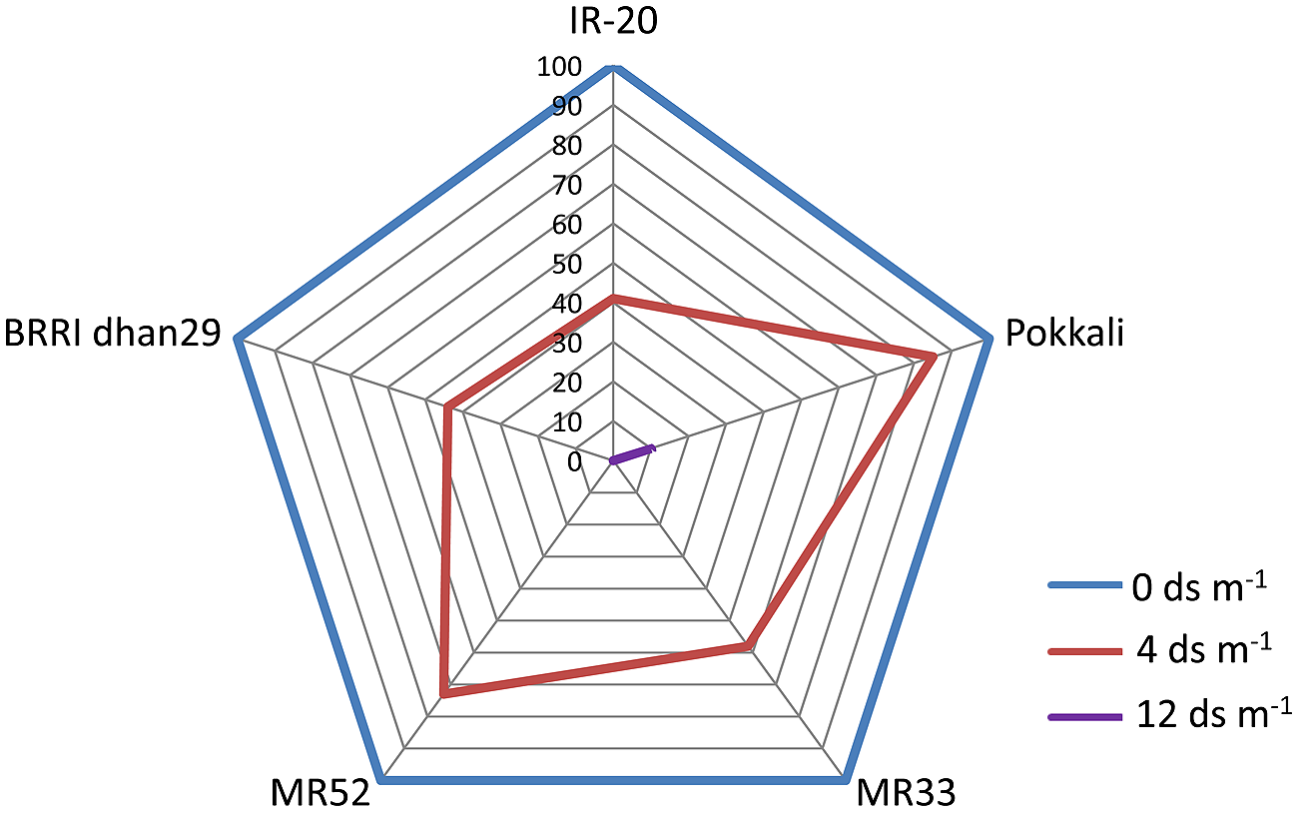
**Percentage decline in yield (g Hill^-1^) of various germplasms of rice (IR-20, Pokkali, MR33, MR52, and BRRI dhan29) in response to salinity.** Web digram was constructed taking yield of each genotype under non-stress condition (0 ds m^-1^) as 100%. Note that Pokkali appears to be most tolerant genotype among the ones studied here, as it could give 10% yield even at 12 ds m^-1^. (Source; Hakim et al., 2014).

Seedling-stage salt tolerance is independent of flowering/reproductive stage tolerance (Singh et al., 2004), and has been established by the behavior of CN499-160-13-6 genotype which is a confirmed susceptible genotype at the juvenile stage but tolerant at the flowering stage. This analysis by Mohammadi-Nejad et al. (2010) indicate that seedling and flowering stage salt tolerance is determined by altogether different set of genes in rice. Recently, another group of rice researcher has analyzed the dry mass of rice shoot and root along with the grain yield under various levels of salinity (Hakim et al., 2014). In their report, it has been shown that the level of salinity is inversely proportional to the rice grain yield (**Figure 1**, **Table 2**). It has also been observed that the dry mass of shoot and root in rice decreases with the increase in the level of salinity. The grain yield reduction in rice by salinity stress might be due to the modification in flexibility of the cell wall, and subsequent reduction in the turgor pressure effectiveness in cell growth (Hakim et al., 2014). However, it is evident that increased salt level in soil disturbs the photosynthesis, causes shrinkage of cell contents, reduces growth and differentiation of tissues, cause imbalance in nutrition, injury of membranes, and ultimately, affects the yield contributing characters (Mahmod et al., 2009; Nejad et al., 2010; Hakim et al., 2014).

**Table 2:** Effect of salinity stress on plant growth as reported for different rice varieties (numbers in the bracket indicate the percentage relative to the control; Source; Hakim et al., 2014).

Rice variety	Salinity level (ds m^-1^)	Shoot Dry wt. (g)	Root Dry wt. (g)
IR-20	0	21.6	2.7
	4	16.4 (76)	1.56 (56)
	12	4.1 (19)	0.57 (20)
Pokkali	0	24.2	2.09
	4	21.2 (87)	1.60 (76)
	12	7.9 (32)	0.85 (41)
MR33	0	2.8	3.28
	4	15.9 (70)	2.28 (69)
	12	6.2 (27)	0.87 (26)
MR52	0	21.0	2.51
	4	16.9 (80)	1.90 (76)
	12	6.1 (29)	0.74 (29)
BRRI dhan29	0	2.9	2.87
	4	14.6 (64)	1.7 (62)
	12	3.9 (17)	0.52 (18)

## Adaptive Mechanisms in Rice for Salinity Tolerance

Under salt stress conditions, rice plants exhibit various mechanisms to overcome the damage such as controlling the seedling vigor, reducing the intake of salt through roots, efficient intra cellular compartmentation and transport of salt.

### Seedling Vigor

Salt stress leads to higher accumulation of Na^+^ in shoots, mainly in mature leaves. Various reports have shown that limiting Na^+^ accretion in shoot part under salt stress is linked to salinity tolerance of barley and wheat (Munns and James, 2003). In rice, it has also been verified that sodium ion accretion in shoot part is comparatively well linked with its growth under salt stress (Yeo et al., 1990). Rice varieties differ considerably in their rate of development with the most vigorous one being the conventional landraces and the shorter ones are the cultivated high yielding varieties. Naturally occurring salt tolerant varieties like Pokkali, Nona Bokora etc. belong to these conventional tall varieties. In spite of having comparable net transport of Na^+^ ion through their roots as partially dwarf salt susceptible cultivars, the high vigor of land races permit them to tolerate growth decline by diluting the Na^+^ content in rice cells.

### Root Permeability and Selectivity

The lethal ions enter into the root along with water that travels from soil to the vascular part of the root by two routes, i.e., symplastic and apoplastic. In apoplastic pathway which is a non-energy driven pathway, water travels through intracellular regions to deliver the salt in xylem. In symplastic pathway, water enters in the roots through epidermal plasma membranes and then travels cell-to-cell through plasmodesmata until discharging to the xylem. Rice is a salinity susceptible crop and it has been revealed that a major quantity of sodium ion transported to the rice shoot parts at the time of salt stress is via apoplastic pathway (Krishnamurthy et al., 2009). Munns (1985) reported that, under 100 mM of sodium chloride stress, the transport rate of Na^+^ ion toward shoots of salt tolerant barley is quite lower (only 20%) in relation to salt sensitive rice plants. This observation indicates that a major involvement of Na^+^ bypass movement in salt stress-induced shoot causes sodium ion accretion in rice shoots.

Although water can passively move from roots through intercellular space, but there are morphological components called as suberin lamellae and Casparian band at the root endo- and exo-dermis, which restricts the apoplastic flow of ions and water to go inside the stele (Schreiber et al., 1999; Enstone et al., 2003). Casparian bands are formed by transverse and radial walls infusing the pores of primary cell wall with aromatic and lipophilic materials and suberin lamellae is deposited to the inside surface of cell walls (Ranathunge et al., 2004). Chemical nature of the root apoplastic barrier is crucial for their performance (Schreiber et al., 1999). It was observed that in roots, apoplastic barriers suberization was most common in salinity tolerant plants, which also has the least Na^+^ accretion in the shoot parts (Krishnamurthy et al., 2009; Cai et al., 2011). Krishnamurthy et al. (2009) have also revealed that in both susceptible and tolerant varieties, the expression of suberin biosynthetic genes was induced under salinity stress, which increased the reinforcement of these barriers in roots of rice. Though the mechanism of apoplastic movement of Na^+^ has not been clear, Na^+^ over-accretion through bypass movement in rice shoots is supposed to be the result of Na^+^ reflexive flow into the xylem. Roots with weak barrier areas like lateral root originating sites and cell walls of root tip area were expected to be the possible entrance sites for Na^+^ bypass movement (Yeo et al., 1987). Ranathunge et al. (2005) reported the disruption of the endodermal Casparian stripes, and ultimately crack through the fence in the exodermis at the time of lateral roots emergence at the pericycle region next to the phloem in the root of monocot. It was also shown that suberin lamella and casparian stripes in both endodermis and exodermis are not detectable at the apices of root (Ranathunge et al., 2003; Schreiber et al., 2005), signifying a weak fence at root apices of rice plants.

**FIGURE 2:**
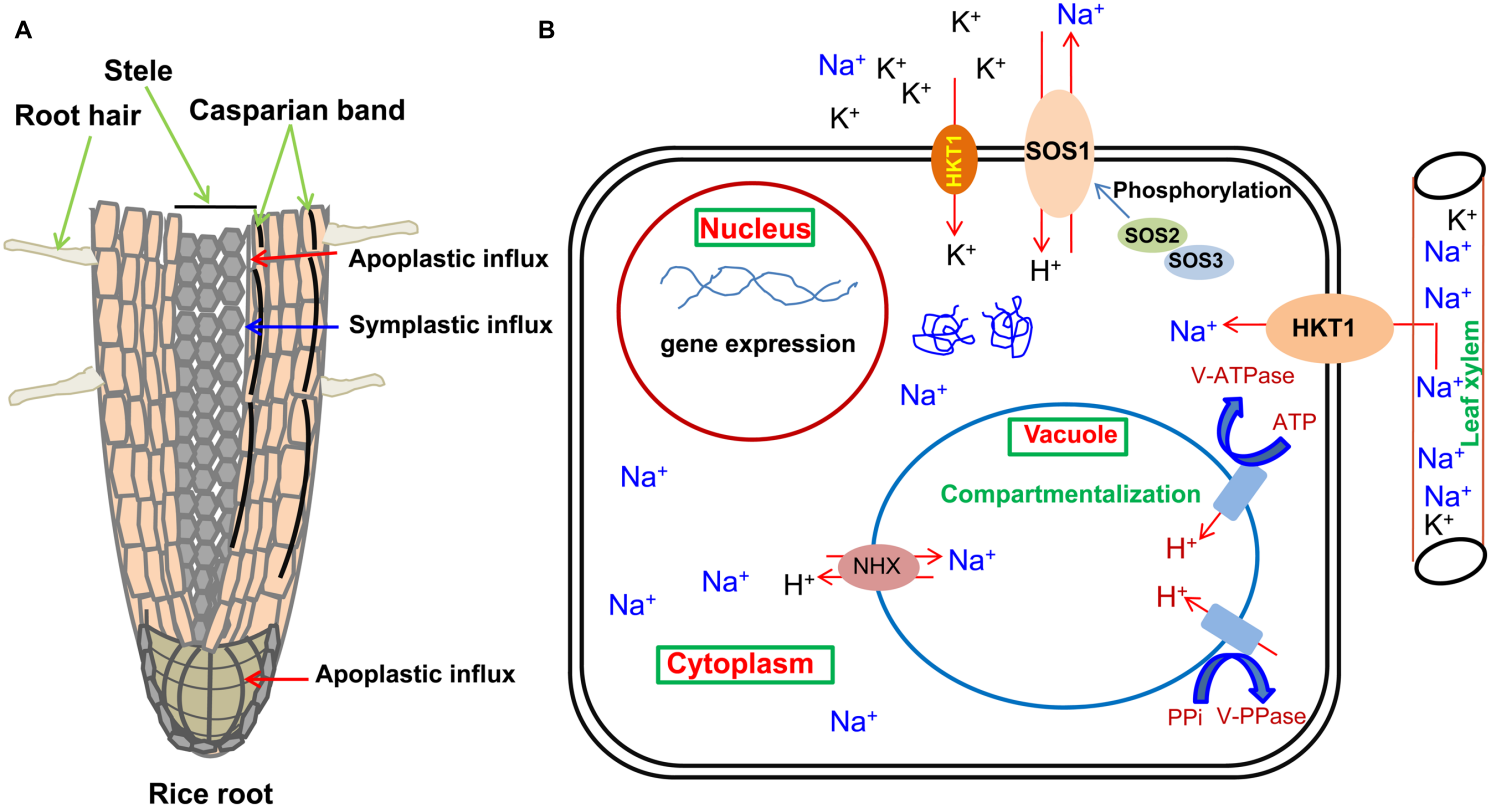
**Schematic representation of Na^+^ influx in roots, its sequestration pathways and primary protective mechanisms as mediated by the transporters present on plasma membrane and tonoplast of the cell. (A)** Influx of Na^+^ through plant root. Red arrows represent probable Na^+^ entry sites for the apoplastic bypass flow and blue arrow represents the path for symplastic movement. **(B)** Various transporters (NHX, HKT, SOS1) responsible for ion movement localized on the biological membranes have been shown for an individual cell of the plant. The energy providing (vacuolar H^+^-ATPase or V-ATPase, vacuolar H^+^-translocating pyrophosphatase or V-PPase) and activating molecules (SOS3, SOS2) are also shown.

Symplastic movement of ions in root involves various ion selective channels/transporters present on the plasma membrane of the root cell which selectively allow the movement of ions inside the cell and maintain ionic balances under salinity. Plants have different defense machinery at the boundary of cell-xylem apoplast. A report has shown that Na^+^ re-intake takes place from the xylem flow by adjacent tissues, and as a consequence, decreases flow of Na^+^ into the shoot parts (Lacan and Durand, 1996). HKT is a Na^+^/K^+^ symporter found in the plant cell membrane which regulates transportation of Na^+^ and K^+^. Class 1 HKT transporter in rice removes excess Na^+^ from xylem, thus protecting the photosynthetic leaf tissues from the toxic effect of Na^+^ (Schroeder et al., 2013). This mechanism of salt tolerance has been depicted in **Figure 2A**.

### Intracellular Compartmentation

Based on osmotic potential, plant can check Na^+^ ion to go into the cell by energy driven process. K^+^ and Na^+^ are interceded by dissimilar transporters which have been verified by Garciadeblás et al. (2003). Cell ion homeostasis is maintained by the ion pumps like symporters, antiporters, and carrier proteins present on the membranes. In cereals, Na^+^ exclusion systems were suggested to be composed of several transporters present on cell membrane like H^+^-pump ATPases, Na^+^/H^+^ antiporter and the high-affinity uptake of K^+^ ion (Jeschke, 1984). Salt Overly Sensitive or SOS pathway of homeostasis is an excellent example of ion management which is turned ‘on’ following the activation of the receptor in response to salinity and transcriptional induction of genes by signaling intermediate compounds (Sanders, 2000). Zhu et al. (1998) first reported three *sos* mutants of *Arabidopsis* which were hypersensitive to specific salt-NaCl. These three *sos1, sos2*, and *sos3* mutants exhibit altered phenotype with reference to Na^+^ accretion. In SOS pathway, calcium binding protein SOS3 directly interacts and activates SOS2, a serine/threonine protein kinase (Liu and Zhu, 1998; Halfter et al., 2000). SOS3 recruits SOS2 on the cell membrane, where SOS2–SOS3 complex phosphorylates SOS1, a Na^+^/H^+^ antiporter on cell membrane, which extrudes Na^+^ out of the cell (Quintero et al., 2002; Guo et al., 2004). Ma et al. (2012) have shown that under salinity stress in *Arabidopsis*, NADPH oxidases also work in ROS-mediated regulation of Na^+^/K^+^ balance.

When higher accumulation of Na^+^ in cytosol occurs, Na^+^ get sequested into the vacuole before it arrives to a toxic point for enzymatic reactions. This pumping action is regulated by vacuolar Na^+^/H^+^ antiporters (Blumwald, 2000). Increase in level of salt induces the Na^+^/H^+^ antiporter action but it amplifies more in salinity tolerant varieties than salinity susceptible ones (Staal et al., 1991). The Na^+^/H^+^ exchange in vacuole is determined through two separate proton pumps, i.e., vacuolar H^+^-ATPase and vacuolar H^+^-translocating pyrophosphatase (Blumwald, 1987). Manipulation in the levels of vacuolar transporter (NHX1) leads to improve salinity tolerance in rice, *Arabidopsis, Brassica* and Tomato (Apse et al., 1999; Zhang et al., 2001; Fukuda et al., 2004). Bassil et al. (2012) have reported one endosomal Na^+^/H^+^ antiporter (OsNHX5) and four vacuolar Na^+^/H^+^ antiporters (OsNHX1-4) in rice (**Figure 2B**).

### Osmoprotectants

Most of the organisms including plants and bacteria accumulate certain organic solutes (such as sugars, proline etc.) due to osmotic stress. These compounds are called osmoprotectants because even when present in high concentrations they do not hinder with cellular enzymatic reactions (Johnson et al., 1968). These are found in cell cytoplasm and the inorganic ions like Cl^-^ and Na^+^ are preferentially seized into the vacuole, consequently leading to the turgor preservation for the cell under osmotic pressure (Bohnert et al., 1995). The non-reducing sugar trehalose possesses a distinctive feature of reversible water storage ability to guard cellular molecules from dehydration stress. Garg et al. (2002) have reported that the trehalose biosynthesis and accumulation in transgenic rice can provide tolerance to salinity and drought stresses. Role of other osmoprotectants such as proline (Ahmed et al., 2010; Deivanai et al., 2011), glycine betaine (Makela et al., 2000; Ahmad et al., 2013), mannitol (Thomas et al., 1995) etc. in salt stress tolerance in plants has also been well documented.

## Approaches for Improving Salinity Tolerance in Rice

### Conventional Methods

Plant breeding methods have been adopted since long time to generate stress tolerant and high yielding rice varieties. Breeders have made genetic alterations in rice crops, at intergeneric, intraspecific and site-specific levels to generate salinity tolerant cultivars. It has been established that source(s) of salt tolerance are still to be explored within the cultivated germplasm of rice (Flowers et al., 1990). Nevertheless, there are evident signals that some conventional rice landraces and varieties (e.g., Pokkali, Bura Rata, and Nona Bokra) are better salinity tolerant than many prominent varieties. Pokkali has been popular as a gene donor in plant breeding programs to develop salinity tolerant cultivars. The better tolerance to salinity in Pokkali is generally credited to both its capacity to preserve low ratio of Na^+^/K^+^ in plants and its quicker expansion rate under salinity. Using IR29 and Pokkali a recombinant inbred population has been produced at the International Rice Research Institute, Philippines (Bonilla et al., 2002). A number of other salt-sensitive and salt-tolerant inbred lines have also been documented during screening for salt tolerance (Gregorio et al., 2002). Many salt tolerant cultivars of rice have been generated in various countries by breeding which includes CSR13, CSR10 and CSR27, IR2151, Pobbeli, PSBRc 84, PSBRc 48, PSBRc 50, PSBRc 86, PSBRc 88, and NSIC 106.

However, the fact is that the wild types or the landraces discussed here are connected with a host of innate difficulties of reduced agronomic characters like photo-sensitivity, tallness, low yield and poor grain quality. Hence, breeding for enhanced salt tolerance using these wild germplasm is a real challenge. Other problem with traditional plant breeding is reproductive difficulty where it is really problematic that if the gene is present in a wild counterpart of the crop, breeder faces trouble in introducing it to the domesticated variety. Therefore, keeping these in mind, several modern approaches have been adopted for production of salinity stress tolerant rice.

### Omics-Based Approaches in the Modern Era

Plant molecular biology seeks to study biological and cellular processes like plant development, its genome organization, and communications with its surroundings. These multi-dimensional detailed studies require large-scale experimentation linking the whole genetic, functional and structural components. These large scale experimentations are known as ‘omics.’ Chief contributors of ‘omics’ include genomics, transcriptomics, proteomics, metabolomics, and phenomics. ‘Omics’ approaches are regularly used in various research disciplines of crop plants, including rice. These approaches have enhanced very fast during the last decade as the technologies advance. Following section describes how ‘omics-based’ approaches have helped in understanding and dissecting out the mechanism of salinity tolerance in rice and helped in generating several salt tolerant germplasms.

#### Genomics-Based Approach

##### Molecular marker resources and quantitative trait loci (QTL) mapping for rice salinity tolerance

Accessibility of the whole genome sequence of rice (Matsumoto et al., 2005) has contributed to the rapid development in the area of functional genomics of salinity tolerance in rice. This information further supported by development of a number of single nucleotide polymorphism (SNP) markers and simple sequence repeat (SSR) markers. Both SSR and SNP marker analysis have been successfully used to discover salt tolerant cultivars of rice (Dhar et al., 2011). In the recent past, development of next generation sequencing (NGS) has enabled the sequencing based genotyping way more efficient (Ray and Satya, 2014). QTL studies for salt stress tolerance have been investigated by several researchers (Bonilla et al., 2002; Gregorio et al., 2002; Lin et al., 2004). Genetic maps of rice have been generated using recombinant inbreed lines developed from genetically distant varieties, such as indica and japonica rice as parents. Such combinations generate appreciably more polymorphism than that between the same subspecies. McCouch et al. (1988) published the first rice genetic map by restriction fragment length polymorphism technique; different fine maps have since been generated using various markers such as amplified SSR, random amplified polymorphic DNA and fragment length polymorphism (Kurata et al., 1994; Harushima et al., 1998). Moreover, the genomic tools [expressed sequence tags (ESTs) from salinity-stressed libraries, expression profiling by microarrays, whole genome sequence information, targeted or random mutation breeding, and complementation and promoter trapping approach] and methods that have become available offer chances to differentiate the salinity-tolerance-likned gene networks in more depth (Bohnert et al., 2001; Kumari et al., 2009; Soda et al., 2013). Seven QTL linked with salinity have been recognized for rice seedlings and mapped to different chromosomes (Prasad et al., 2000). Using F2 population obtained from a salinity tolerant mutant of rice (M-20) and the salinity susceptible wild variety (77–170A), a key gene for salinity tolerance has been mapped on chromosome 7 (Zhang et al., 1995). Koyama et al. (2001) showed the chromosomal location selectivity traits of an ion transport which are companionable with agronomic demands. Gregorio et al. (2002) have mapped a major *Saltol* QTL which is flanked by markers RM23 and RM140 on chromosome 1, using a population raised from a cross among Pokkali and IR29. More than 70% of the difference in salt uptake has been accounted by this QTL (Bonilla et al., 2002). Pokkali was the basis of positive alleles for this QTL, which accounted for decreased sodium and potassium ratio under salinity (Bonilla et al., 2002; Gregorio et al., 2002). Lin et al. (2004) have shown a QTL for increased shoot K^+^ under salt stress in the similar position of chromosome 1. Mapping SKC1 on chromosome 1 was a breakthrough which preserves K^+^ ion homeostasis in the salinity-tolerant cultivar (Nona Bokra) under salinity conditions (Ren et al., 2005).

##### Introduction of desired gene/genes into the rice genome for salinity tolerance – the ‘reverse-genetic’ approach

Plants react to salinity by limiting the intake of toxic ions like Na^+^ and regulate their osmotic potential by producing compatible solutes (sugars, glycinebetaine, proline etc.) and partitioning toxic ions into the tonoplasts to maintain low Na^+^ levels in the cytoplasm (Blumwald and Grover, 2006). Salinity tolerant transgenic rice plants were generated by getting ideas from the above observation (Kumar et al., 2013). Xu et al. (1996) produced transgenic rice by introduction and over-expression of late embryogenesis abundant (LEA) protein from barley. Their study demonstrated that the transgenic rice possessed a better growth rate under 200 mM of salinity and better recovery upon removal of stress. Similarly, genetically engineered rice has also been developed with the capacity to produce glycinebetaine by a gene (*codA*) encodes choline oxidase and it has been found to have better salt (150 mM NaCl) tolerance than the WT (Mohanty et al., 2002). Transgenic rice plants developed by over-expressing OsCDPK7 (a calcium-dependent protein kinase) gene were found to have the youngest leaf drooped 3 days after treatment with 200 mM sodium chloride in wild type plants, whereas transgenic plants showed better tolerance (Saijo et al., 2000). Several latest reports have shown a host of other genes related to antioxidants, transcription factors, signaling, ion homeotasis and transporters found to have key role in salinity tolerance (Garg et al., 2002; Blumwald and Grover, 2006; Zhao et al., 2006a; Jiang et al., 2012; Kumar et al., 2012; Kumari et al., 2013; Liu et al., 2014a; Rachmat et al., 2014).

##### Genome modification through mutation breeding for salinity tolerance in rice – the ‘forward-genetics’ approach

Although efforts to advance stress tolerance in plant by genetic manipulation have resulted in some significant achievements, mutation breeding technique has been accepted as a foremost strategy to obtain stress tolerant varieties as well as varieties with other desired traits (Wang et al., 2003; Flowers, 2004). Mutation breeding has a significant contribution toward production of high yielding and salt stress tolerant rice varieties (Cassells and Doyle, 2003; Parry et al., 2009; Das et al., 2014). There are many reports where mutation breeding has resulted in enhanced salinity tolerance in various rice cultivars. For example, rice seeds irradiated with carbon (C) or neon (Ne) ions have generated mutant variety with high salt tolerance (Hayashi et al., 2007). The Azolla-Anabaena symbiotic system provides green manure for flooded crops, mainly rice. Mutation breeding has produced Azolla variants tolerant to high salinity, toxic aluminum levels, and to herbicides (Novak and Brunner, 1992). Many such varieties of salinity tolerant mutant rice have been released in many countries all over the world so far and some of them have been listed in **Table 3**.

**Table 3:** Salinity tolerant rice varieties produced through mutation breeding.

Crop variety	Mutation technique	References
Rice (6 B)	γ irradiation	Singh (2000)
Rice (A- 20)	γ irradiation	Singh (2000)
Rice (Atomita 2)	γ irradiation	Singh (2000)
Rice (Changwei 19)	γ irradiation	Singh (2000)
Rice (Emai No. 9)	γ irradiation	Singh (2000)
Rice (Fuxuan No. 1)	γ irradiation	Singh (2000)
Rice (Liaoyan 2)	γ irradiation	Singh (2000)
Rice (Mohan = CSR 4)	γ irradiation	Singh (2000)
Rice (Jiaxuan No. 1)	γ irradiation	Singh (2000)
Rice (Nipponbare)	γ irradiation	Hayashi et al. (2008)
Rice	γ irradiation	Jain et al. (1998), Jain and Suprasanna (2011)
Rice (Niab-irri-9)	γ irradiation	www.niab.org.pk/
Rice (Shua 92)	γ irradiation	Baloch et al. (2003)
Rice (Basmati 370)	γ irradiation	Saleem et al. (2005)

#### Transcriptomics Approach

Transcriptomics, also called as expression profiling, generally require a systematic and entire study of all the RNA transcripts that signifies the spatial and temporal gene expression of a cell, tissue of an organism under a certain biological circumstance (Thompson and Goggin, 2006). This technique leads to identification of a large number of differentially regulated transcripts due to cross talks and overlapping pathways under particular stress/environmental situations (Sahi et al., 2006; Walia et al., 2007). Microarrays have become one of the standard tools in molecular biology and have taken as commanding approach for the analysis of genome wide transcriptional response by studying the expression of all the expressed genes in a single experiment. The complete transcriptome at a given time point allow us to detect any stress-inducible genes which can suggest the specific biological processes and/or the regulation of transcriptional and translational machineries that are induced (Gracey and Cossins, 2003). In rice, EST based cDNA arrays and oligonucleotide microarrays have been used to understand the underlying biological meaning by studying and comparing the global gene expression patterns (Eyidogan and Öz, 2007). In the recent past, stress (including salinity)-inducible transcripts in rice were identified by using microarray technology (Wilson et al., 2007; Kumari et al., 2009; Plett et al., 2011; Garg et al., 2013). It is well documented that the mechanisms involved in salinity tolerance is complex and polygenic trait (Munns and Tester, 2008). Introduction of a single gene is least likely to improve the salt tolerance considerably. As an alternative, multiple genes involved in the key mechanism of the processes such as signaling, osmotic adjustment, ion homeostasis, vacuolar compartmentalisation of ions, restoration of enzymatic activity and oxygen free radical scavenging should be used (Bohnert et al., 2001). The transcription factors having a cascade effect that can regulate many other downstream genes may also prove vital in this regard. The main challenge is that it is not yet clear what are the genes that are needed to be studied and manipulated (Cuartero and Bolarin, 2010). Salt tolerance in rice is advantageous in this regard as rice is particularly salt sensitive at seedling and reproductive phase and a few QTLs having large effects are known to control the trait (Leung, 2008). The traits, though, have low heritability and are usually inherited quantitatively (Cuartero et al., 2006). The measurements of these traits in segregating populations are not always easy which demands careful coordination of environmental conditions over seasons and locations. The capability to evaluate the expression levels of whole genome in a single experiment by microarray technique allows biologists to see what are the genes induced or repressed under specific environmental extremes. The constraint is that in addition to the actual genes that control the stress response, it detects enormous number of related genes which might be involved in secondary or irrelevant downstream functions (Cuartero et al., 2006). Beside the challenge of recognizing the relevant target genes, the transcriptomic approach offers an efficient tool of identifying the gene(s) involved in specific stress tolerance mechanism.

#### Proteomics Approach

The study of a protein is the shortest and direct way to describe the role of the gene linked with the particular protein. Nevertheless, it must be noted that the proteome and genome of an organism do not always communicate to each other directly (Komatsu et al., 2009). Thus, the investigation done at the metabolome and proteome levels are evenly significant as the study of genomics. Proteomics study gives a platform to analyze complex biological functions which includes huge numbers of proteins as well as the interacting network of various proteins. Proteomics can serve as a main technique for exposing the molecular machineries that are concerned in interactions among the plant and diverse stresses including salt stress. Salinity stress induces the expression of various genes which are eventually reflected in the profile of the proteins. The function of salinity- and other stress-induced proteins has been extended by proteomic study in different tissue parts of rice (Fukuda et al., 2003; Chen et al., 2009; Lee et al., 2009; Liu and Bennett, 2011).

A scheme for direct recognition of proteins by differential display approach has been established and the proteins structure can be recognized by evaluation with the proteome database of rice or by Edman sequencing and mass spectrometry (MS). It has been shown that the present rice proteomic studies have so far concentrated on the recognition of polypeptides based on their available quantity upon subjecting to different stresses (Ma et al., 2013). The complex physiological response data of proteomics study were found to change over the severity of stress, therefore, making the data evaluation and integration analyses complicated. Henceforth, post-translational modification (PTM) could be a substitute to examine stress signaling functions. Many strategies have been created to distinguish PTM in plants. Particularly, the use of two dimensional PAGE method joined with the application of 5′-iodoacetamidofluorescein (5′IAF) or 2D-fluorescence difference gel electrophoresis (DIGE) allows the recognition of oxidized and reduced stress-linked proteins (Cuddihy et al., 2008; Fu et al., 2008). Several gel-free methods have also been identified for differential analysis of proteome. Instances are: multidimensional protein identification method that successfully recognizes individual protein components by eliminating band broadening for chromatographic recognition (Koller et al., 2002), isobaric tags for relative and absolute quantification and isotope-coded affinity tags (Griffin et al., 2001). These methods are considered as targeted techniques to recognize alteration in proteins by mass difference mean, among different proteomes.

#### Metabolomics Approach

Metabolites are the final product of cellular reactions which reflect the reaction of biological systems to environmental fluctuations (Royuela et al., 2000). The present movement in metabolomic analysis is to describe the cellular position at a specific stage by assessment of the whole metabolites in the cell (Hollywood et al., 2006). Metabolomics techniques complement proteomics and transcriptomics technique and show exact figures of the whole cellular course. A sequence of investigative method is accessible for the study of plant metabolome (Okazaki and Saito, 2012), along with the application of modern and high throughput methods such as Fourier transform infrared (FT-IR; Johnson et al., 2003), ultra high-resolution fourier transform-ion cyclotron MS (Hirai et al., 2004), gas chromatography-MS (GC-MS; Kaplan et al., 2004), and nuclear magnetic resonance (NMR; Kim et al., 2010). Metabolomics came into view as an important tool for the study of environmental responses in plants (Bundy et al., 2009). Rice metabolomics studies have so far determined the types and the quality of metabolites which can endorse the germination of seed (Shu et al., 2008), the variation of metabolites among wild type and mutant plants (Wakasa et al., 2006), the metabolome profiling at various stages of development (Tarpley et al., 2005), and the examination of natural metabolite dissimilarity among different rice varieties (Kusano et al., 2007). Few studies have shown the metabolic impact of salinity on crop plants such as rice, tomato, grape vine, *Solanum lycopersicon* and *Arabidopsis* (Johnson et al., 2003; Gong et al., 2005; Zuther et al., 2007). Plants react to adverse situations by a sequential modification of their metabolism with transient, sustained, early reactive and late reactive metabolic changes. For instance, proline and raffinose gather to increased levels upon several days of exposure to salinity, cold, or drought, while central carbohydrate metabolism changes quickly in a time-dependent and complex way (Kaplan et al., 2004; Urano et al., 2009; Lugan et al., 2010). Some metabolic alterations are common to all the abiotic stress types, but others are particular. For instance, levels of sugars, sugar alcohols and amino acids usually amplify with response to different stresses. Remarkably, proline accumulates upon salinity, drought and cold stress but not upon heat stress (Kaplan et al., 2004; Gagneul et al., 2007; Kempa et al., 2008; Usadel et al., 2008; Urano et al., 2009; Lugan et al., 2010). In most of the studies, amount of TCA-cycle intermediates and organic acids got declined in glycophytes after salinity stress (Gong et al., 2005; Zuther et al., 2007; Gagneul et al., 2007), but got enhanced upon drought or temperature stress (Kaplan et al., 2004; Usadel et al., 2008; Urano et al., 2009). Usually, sugars are essential compatible solutes gathered in cells during stress response. Fumagalli et al. (2009) studied the metabolite profile of two different cultivars (Nipponbare and Arborio) of rice under salinity (150 mM) and showed enhanced sugar contents during salinity stress in both the cultivars. Their results also showed that salt stress altered the accumulation of various metabolites (glutamate, aspartate, proline, valine, lactate, alanine, malate etc.) in rice which have vital role in salt tolerance. They also suggested that NMR coupled with principal component analysis (PCA) is a commanding tool to characterize rice varieties under salinity or any other stress.

#### Phenomics Approach

Plant phenomics is advanced screening method which includes the study of plant phenotype, growth, and performance and eventually, identification of the required trait. Couple of screening methods for various morpho-physiological traits have been used to measure the tolerance to salinity in rice, including plant weight, Na^+^ concentration, the ratio of Na^+^/K^+^ in shoot, leaf injury, survival rate of leaf following injury, leaf area and bypass movement in the root (Yeo et al., 1990; Asch et al., 2000; Zeng et al., 2003; Faiyue et al., 2012). However, most protocols that measure plant biomass are destructive, therefore making it difficult to measure active responses in plant growth in response to salt application and to collect seed from the individuals being measured. Current progress in image-based phenotyping have enabled the non-destructive assessment of plant responses to salinity over time and allow determination of shoot biomass measurements without having to harvest the whole plant (Rajendran et al., 2009; Berger et al., 2012; Hairmansis et al., 2014; Jansen et al., 2014). Upon salinity stress, growth of rice plants immediately slows due to stress, and plants produce fewer tillers (Munns and Tester, 2008; Rajendran et al., 2009; Horie et al., 2012). Over time, Cl^-^ and Na^+^ accumulate to lethal concentrations in the plant, resulting in premature senescence of leaf and subsequent death (Munns and Tester, 2008; Munns et al., 2010; Horie et al., 2012). Notably, image-based phenotyping can differentiate among the effects of the osmotic and ionic components of salt stress in growing plants. It can be done by growth response measurement immediately after application of salt, before the increase in accumulation of toxic ions in the shoot. This permits for at least some analysis of salinity tolerance mechanisms (Rajendran et al., 2009; Sirault et al., 2009). A non-destructive image-based phenotyping method to analyze the responses of rice to different levels of salinity stress has been developed and revealed differences in the effects of salinity in two cultivars of rice, IR64 and Fatmawati (Hairmansis et al., 2014).

Automation of the phenotyping process in combination with automated plant handling and watering allows large numbers of plants to be screened efficiently with short handling. Entire populations of plant can be grown in soil media, emulating field conditions (at least for the earlier growth stages), hence permitting the transfer of knowledge from controlled environment to field growth conditions. Screening of 100s of mapping lines and/or rice accessions for bi-parental or association mapping studies can now be done relatively quickly for traits that require time course growth assessments. The use of these populations has the prospective to reveal the underlying genetic mechanisms of salinity tolerance in a forward genetics screen. As costs decrease, so the power of this approach will also increase, to allow more detailed characterization of rice genotypes (e.g., stomatal behavior) in response to salinity [e.g., by combination of infrared (IR), Red–Green–Blue (RGB) imaging and fluorescence techniques]. Use of non-destructive imaging technologies, in combination with measurements of tissue ion concentration, allows the differentiation between the osmotic and ionic components of salt stress in rice. This will allow the detection of new traits and sources of salinity tolerance genes that can be used to pyramid different salinity tolerance mechanisms into elite rice breeding lines.

### Integration of ‘Omics’ Approach

**FIGURE 3:**
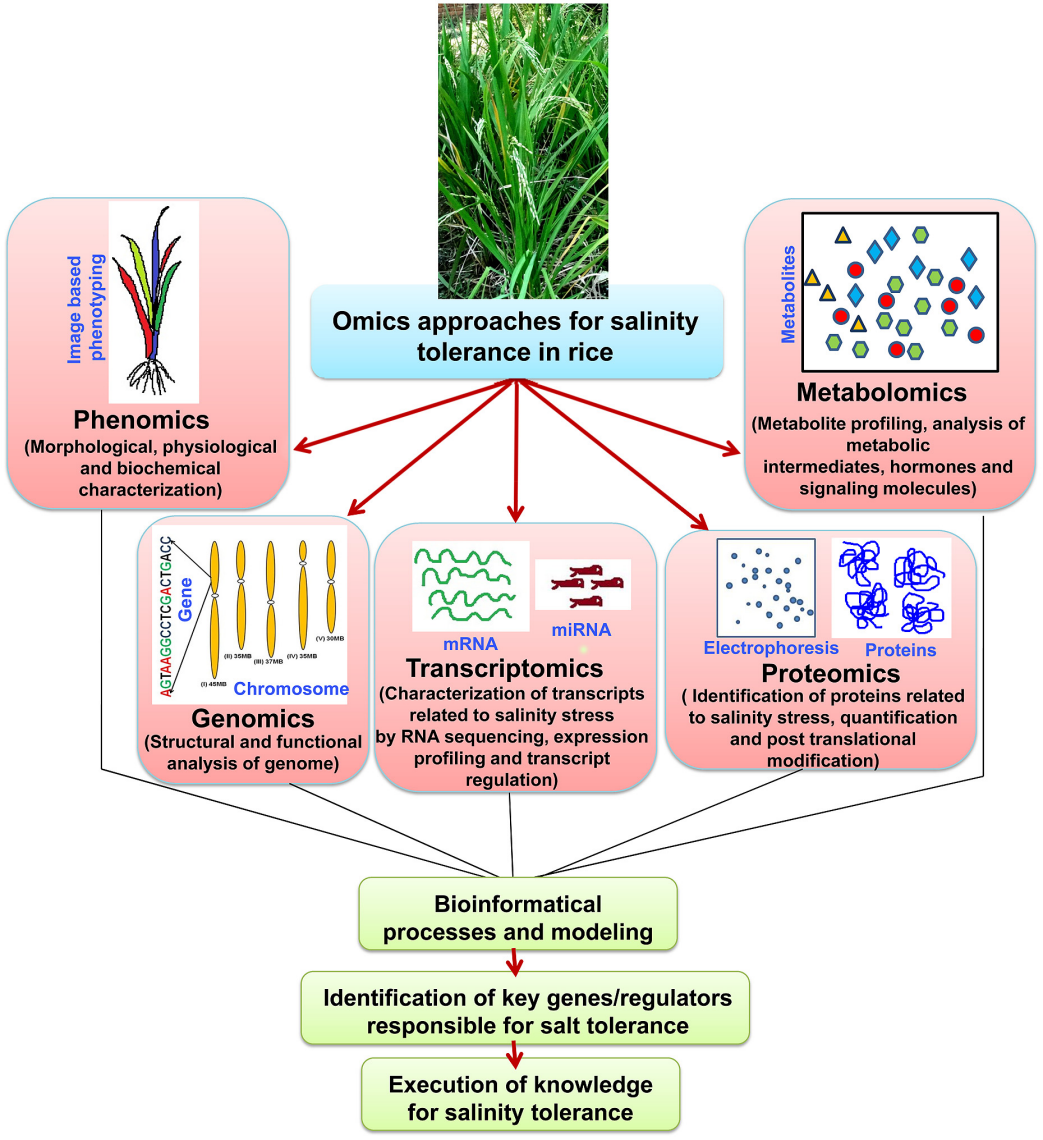
**‘Omics-based’ approaches and their integration to develop salinity tolerant rice cultivars.** Flow of information from various ‘omics-based’ platforms such as phenomics, genomics, transcriptomics, proteomics, and metabolomics needs to be integrated to understand complex traits such as salinity tolerance. Ultimately, the key genes/regulators responsible for salt tolerance need identification and validation using the tools of functional genomics.

‘Omics’ approaches seems to be overlapping and dependent on each other, and integration of all the ‘omics’ approaches is necessary to reach at an ultimate step i.e., raising of stress tolerant cultivars (**Figure 3**). Proteomic studies show vast overlapping in vital metabolism (e.g., Calvin cycle and carbohydrate metabolism) under salt stress. However, different metabolic pathways have been found to control and regulate under metabolomic level, mostly the biosynthesis of amino acid, photorespiration and citric acid pathway (Ma et al., 2013). This is probably due to the participation of downstream enzymatic reaction instead of cellular injury reactions, which encourage the pathways of complex metabolites. The proteomic approach presumes that the raise in quantity of protein amount is always escorted by biologically active compound, but in fact it may include the factors by posttranslational alterations, that may alter characteristics of the proteins. So, the function of the metabolite changing occured at the metabolomic point is not very much clear. Hence, it can be said that the growth of bioinformatics, in linking to the response at transcription level to either metabolomic or proteomic alterations, is yet to be done. The quick evolution in ‘omics’ research has led to more and more generation of data sets throughout all branches in life science studies. Different investigative applications, that are vital for the efficient incorporation of data resources, have been published in various databases. These huge datasets are found via four main stages: (a) data generation (b) data processing (c) data integration and the final step is (d) data analysis (Mochida and Shinozaki, 2011). The dispute in the incorporation of omics data investigation has been argued (Edwards and Batley, 2004). It was shown that the main trouble occurred from the partial and dissimilar form of information accessible on bioinformatics data sources. Hence, algorithmic methods have been planned as the solution for this kind of trouble (Ge et al., 2003). Currently, many servers have been established which allows the integration of high-throughput data and these servers also able to display the outcomes in a meaningful pathway of biology (Tuncbag et al., 2012).

The ultimate aim of integration of ‘omics-based’ approaches is to find out key stress responsive genes/proteins and introduction of those genes/proteins for generation of improved stress tolerant crop varieties. A list of transgenic salt tolerant rice cultivars generated by the introduction of key salt responsive genes/proteins has been listed in **Table 4**.

**Table 4:** Transgenic rice cultivars developed by introduction of genes/proteins identified through ‘omics-based’ approach.

Source organism	Gene/Protein	Target rice cultivar	Reference
*Oryza sativa*	Glutamine synthetase	Kinuhikari	Hoshida et al. (2000)
*Avena sativa*	Arginine decarboxylase	TNG-67	Roy and Wu (2001)
*Arthrobacter globiformis*	Choline oxidase	Pusa Basmati-1	Mohanty et al. (2002)
*Hordeum vulgare*	LEA protein	Pusa Basmati 1	Rohila et al. (2002)
*Atriplex gmelini*	Vacuolar Na^+^/H^+^ antiporter	Kinuhikari	Ohta et al. (2002)
*O. sativa*	MAP kinase	Nipponbare	Xiong and Yang (2003)
*Escherichia coli*	Trehalose -6- phosphate synthase and Trehalose -6- phosphate phosphatase	Nakdong	Jang et al. (2003)
*Vigna aconitifolia*	Δ1-pyrroline-5-carboxylate synthetase	Kenfong	Su and Wu (2004)
*O. sativa*	Vacuolar Na^+^/H^+^ antiporter	Nipponbare	Fukuda et al. (2004)
Mouse	Calcineurin	Xiushui 04	Ma et al. (2005)
*Arabidopsis thaliana*	MYB transcription factor	TNG-67	Malik and Wu (2005)
*Suaeda salsa*	Catalase	Zhonghua No. 11	Zhao and Zhang (2006)
*A. thaliana* and *O. sativa*	DREB transcription factor	Kita-ake	Ito et al. (2006)
*O. sativa*	NAC transcription factor	Nipponbare	Hu et al. (2006)
*Suaeda salsa*	Vacuolar Na^+^/H^+^ antiporter	Zhonghua-11	Zhao et al. (2006b)
*Schizosaccharo- myces pombe*	Plasma membrane Na^+^/H^+^ antiporter	Zhonghua-11	Zhao et al. (2006a)
*Arthrobacter pascens*	Choline oxidase	TNG-67	Su et al. (2006)
*Avicennia marina*	SOD	Pusa Basmati-1	Prashanth et al. (2008)
*E. coli*	Catalase	Nipponbare	Nagamiya et al. (2007)
*O. sativa*	Glyoxalase II	Pusa Basmati-1	Singla-Pareek et al. (2008)
*Pennisetum glaucum*	Vacuolar Na^+^/H^+^ antiporter	Pusa Basmati-1	Verma et al. (2007)
*O. sativa*	Shaker potassium channel	Nipponbare	Obata et al. (2007)
*E. coli*	Catalase	Nipponbare	Motohashi et al. (2010)
*O. sativa*	*Ribosome*-*inactivating protein gene 18*	Nipponbare	Jiang et al. (2012)
*O. sativa*	Calmodulin-like gene	Pei’ai 64S	Xu et al. (2013)
*O. sativa*	Heat shock transcription factor *A7*	*Nipponbare*	Liu et al. (2013)
*O. sativa*	Vesicle trafficking gene	Zhonghua 11	Peng et al. (2014)
*O. sativa*	bZIP transcription factor	Zhonghua 11	Liu et al. (2014b)
Bermudagrass	DREB transcription factor	Jonghua 11	Chen et al. (2015)
*Citrus tristeza virus*	*Heat shock protein 70*	*Nipponbare*	Hoang et al. (2015)
*A. thaliana*	Bcl-2 associated gene product	*Nipponbare*	Hoang et al. (2015)
Baculovirus	*p35*	*Nipponbare*	Hoang et al. (2015)

## Conclusion

The world population is increasing with fast pace supplemented with reducing cultivable land due to salinization of arable land naturally or by improper irrigation practices. Altogether, this leads to decrease in the production of salt sensitive cereal crop rice, a staple food grain of developing world. Presently, a lot of methods have been implicated for modifying the genetic makeup of rice plant to withstand high salinity and lesser yield compromise. Plant breeding and genetic engineering are two major adopted methods.

Plant breeding is an important tool for crop improvement to develop environmental stress tolerant crops and several salinity tolerant varieties for diverse rice plants were developed till the present date. Nevertheless, this technology has its own limitations on which it is based i.e., reproductive obstacle and fine genetic variations of rice. However, mutation breeding has come up as a robust tool to substitute the traditional breeding method. Genetic engineering and mutation breeding have effectively used the genetic alterations available for salinity tolerance in the wild counterparts as well as in other organisms for the generation of salt tolerant rice. Although, genome sequencing of rice plant was completed a decade back, but the function of a large group of genes is not known yet. Not only from rice, many genes of unidentified role (20–30% in each genome sequenced) from other plants can convey multiple stress tolerance and can be used for raising salinity tolerant transgenic rice. There is also lack of the integration of information from genomic, transcriptomic, proteomic, metabolomic as well phenomic studies, which is very important for the determination of key pathways or processes involved in complex trait like salinity tolerance. Additionally, even after significant development in the understanding of responses of plant stress, there is still a huge gap in our understanding of sensor and receptor in the signal transduction, signaling molecules and ion transporter. The use of “omics” tool along with genetic engineering and mutation breeding techniques have promising role in delineating gene response and gene function in plants under salinity stress.

Salt tolerance is a multigenic trait, which involves a complex of responses at metabolic, cellular, molecular, physiological and whole-plant levels. Till date, scientists around the world have developed a number of salt tolerant transgenic rice by altering genes involved in various salinity reaction mechanisms such as ion transport and balance, hormone metabolism, osmotic regulation, antioxidant metabolism, and stress signaling. In spite of successful raising of transgenic rice for salinity tolerance in plants, attainment has not been realized at field level yet. The future focus should be on in-depth study of intercellular and intracellular molecular interactions involved in salinity stress response and genetic engineering with key genes coding components of salt tolerance machinery in rice. Last but not the least, the salt tolerant transgenic rice should be in the hand of final user i.e., farmer.

## Author Contributions

AP and SLS-P designed the concept of the manuscript. PD and KN performed the analysis of the topic, and drafted the figures, tables and manuscript. All authors read and approved the final manuscript.

## Conflict of Interest Statement

The authors declare that the research was conducted in the absence of any commercial or financial relationships that could be construed as a potential conflict of interest.

## References

[B1] Ahmad R. Lim C. J. Kwon S. Y. (2013). Glycine betaine: a versatile compound with great potential for gene pyramiding to improve crop plant performance against environmental stresses. *Plant Biotech. Rep.* 7 49–57. 10.1007/s11816-012-0266-8

[B2] Ahmed C. B. Rouina B. B. Sensoy S. Boukhriss M. Abdullah F. B. (2010). Exogenous proline effects on photosynthetic performance and antioxidant defense system of young olive tree. *J. Agricult. Food Chem.* 58 4216–4222. 10.1021/jf9041479 20210359

[B3] Akbar M. (1986). “Breeding for salinity resistance in rice,” in *Prospects for Bio-saline Research* eds Ahmed R. Pietro A. S. (Karachi: University of Karachi) 37–55.

[B4] Akbar M. Yabuno T. (1974). Breeding for saline resistant varieties of rice. Inheritance of delayed type panicle sterility induced by salinity. *Jpn. J. Breed.* 27 237–240. 10.1270/jsbbs1951.27.237

[B5] Apse M. P. Aharon G. S. Snedden W. A. Blumwald E. (1999). Salt tolerance conferred by overexpression of a vacuolar Na^+^/H^+^ antiporter in *Arabidopsis. Science* 285 1256–1258. 10.1126/science.285.5431.1256 10455050

[B6] Asch F. Dingkuhn M. Dörﬄing K. Miezan K. (2000). Leaf K+/Na+ ratio predicts salinity induced yield loss in irrigated rice. *Euphytica* 113 109–118. 10.1023/A:1003981313160

[B7] Baloch A. W. Soomro A. M. Javed M. A. Bughio H. R. Alam S. M. Bughio M. S. (2003). Induction of salt tolerance in rice through mutation breeding. *Asian J. Plant Sci.* 2 273–276. 10.3923/ajps.2003.273.276

[B8] Barrett-Lennard E. G. (2002). Restoration of saline land through revegetation. *Agric. Water Manag.* 53 213–226. 10.1016/S0378-3774(01)00166-4

[B9] Bassil E. Coku A. Blumwald E. (2012). Cellular ion homeostasis emerging roles of intracellular NHX Na+/H+ antiporters in plant growth and development. *J. Exp. Bot.* 63 5727–5740. 10.1093/jxb/ers250 22991159

[B10] Berger B. de Regt B. Tester M. (2012). “High-throughput phenotyping of plant shoots,” in *High-Throughput Phenotyping in Plants. Methods in Molecular Biology* ed. Normanly J. (New York, NY: Humana Press) 9–20. 10.1007/978-1-61779-995-2_2 22893282

[B11] Blumwald E. (1987). Tonoplast vesicles as a tool in the study of ion transport at the plant vacuole. *Physiol. Plant.* 69 731–734. 10.1111/j.1399-3054.1987.tb01993.x

[B12] Blumwald E. (2000). Sodium transport and salt tolerance in plants. *Curr. Opin. Cell Biol.* 12 431–434. 10.1016/S0955-0674(00)00112-5 10873827

[B13] Blumwald E. Grover A. (2006). “Salt tolerance,” in *Plant Biotechnology: Current and Future uses of Genetically Modified Crops* ed. Halford N. G. (Chichester: John Wiley and Sons Ltd.) 206–224. 10.1002/0470021837.ch11

[B14] Bohnert H. J. Ayoubi P. Borchert C. Bressan R. A. Burnap R. L. Cushman J. C. (2001). A genomics approach towards salt stress tolerance. *Plant Physiol. Biochem.* 39 295–311. 10.1016/S0981-9428(00)01237-7

[B15] Bohnert H. J. Nelson D. E. Jensen R. G. (1995). Adaptations to environmental stresses. *Plant Cell* 7 1099–1111. 10.1105/tpc.7.7.1099 12242400 PMC160917

[B16] Bonilla P. Dvorak J. Mackill D. Deal K. Gregorio G. (2002). RFLP and SSLP mapping of salinity tolerance genes in chromosome 1 of rice (*Oryza sativa* L.) Using recombinant inbred lines. *Philipp. J. Agric. Sci.* 85 68–76.

[B17] Bundy J. G. Davey M. P. Viant M. R. (2009). Environmental metabolomics: a critical review and future perspectives. *Metabolomics* 5 3–21. 10.1007/s11306-008-0152-0

[B18] Cai X. Chen T. Zhou Q. Y. Xu L. Qu L. Q. Hua X. J. (2011). Development of Casparian strip in rice cultivars. *Plant Signal. Behav.* 6 59–65. 10.4161/psb.6.1.13545 21248477 PMC3122007

[B19] Cassells A. C. Doyle B. M. (2003). Genetic engineering and mutation breeding for tolerance to abiotic and biotic stresses: science, technology and safety. *Bulg. J. Plant Physiol. Special Issue* 52–82.

[B20] Chen M. Zhao Y. Zhuo C. Lu S. Guo Z. (2015). Overexpression of a NF-YC transcription factor from bermudagrass confers tolerance to drought and salinity in transgenic rice. *Plant Biotechnol. J.* 13 482–491. 10.1111/pbi.12270 25283804

[B21] Chen X. Wang Y. Li J. Jiang A. Cheng Y. Zhang W. (2009). Mitochondrial proteome during salt stress-induced programmed cell death in rice. *Plant Physiol. Biochem.* 47 407–415. 10.1016/j.plaphy.2008.12.021 19217306

[B22] Chinnusamy V. Jagendorf A. Zhu J. K. (2005). Understanding and improving salt tolerance in plants. *Crop Sci.* 45 437–448. 10.2135/cropsci2005.0437

[B23] Cuartero J. Bolarin M. C. (2010). “Molecular tools for enhancing salinity tolerance in plants,” in *Molecular Techniques in Crop Improvement* eds Jain S. M. Brar D. S. (Berlin: Springer).

[B24] Cuartero J. Bolarin M. C. Asíns M. J. (2006). Increasing salt tolerance in the tomato. *J. Exp. Bot.* 57 1045–1058. 10.1093/jxb/erj102 16520333

[B25] Cuddihy S. L. Baty J. W. Brown K. K. Winterbourn C. C. Hampton M. B. (2008). Proteomic detection of oxidized and reduced thiol proteins in cultured cells. *Methods Mol. Biol.* 519 363–375. 10.1007/978-1-59745-281-6_23 19381595

[B26] Das P. Mishra M. Lakra N. Singla-Pareek S. L. Pareek A. (2014). “Mutation breeding: a powerful approach for obtaining abiotic stress tolerant crops and upgrading food security for human nutrition,” in *Mutagenesis: Exploring Novel Genes and Pathways* eds Tomlekova N. Kojgar I. Wani R. (Wageningen: Wageningen Academic Publisher) 15–36.

[B27] Deivanai S. Xavier R. Vinod V. Timalata K. Lim O. F. (2011). Role of exogenous proline in ameliorating salt stress at early stage in two rice cultivars. *J. Stress Physiol. Biochem.* 7 157–174.

[B28] Dhar R. Sägesser R. Weikert C. Yuan J. Wagner A. (2011). Adaptation of *Saccharomyces cerevisiae* to saline stress through laboratory evolution. *J. Evol. Biol.* 7 1135–1153. 10.1111/j.1420-9101.2011.02249.x 21375649

[B29] Dionisio-Sese M. L. Tobita S. (2000). Effects of salinity on sodium content and photosynthetic responses of rice seedlings differing in salt tolerance. *J. Plant Physiol.* 157 54–58. 10.1016/S0176-1617(00)80135-2

[B30] Edwards D. Batley J. (2004). Plant bioinformatics: from genome to phenome. *Trends Biotechnol.* 22 232–237. 10.1016/j.tibtech.2004.03.002 15109809

[B31] Enstone J. E. Peterson C. A. Ma F. S. (2003). Root endodermis and exodermis: structure, function, and responses to the environment. *J. Plant Growth Regul.* 21 335–351. 10.1007/s00344-003-0002-2

[B32] Eyidogan F. Öz M. T. (2007). Effect of salinity on antioxidant responses of chickpea seedlings. *Acta Physiol. Plant.* 29 485–493. 10.1007/s11738-007-0059-9

[B33] Faiyue B. Al-Azzawi M. J. Flowers T. J. (2012). A new screening technique for salinity resistance in rice (*Oryza sativa* L.) seedlings using bypass flow. *Plant Cell Environ.* 35 1099–1108. 10.1111/j.1365-3040.2011.02475.x 22171658

[B34] FAO. (2009). *FAO Statistical Databases.* Available at: http://faostat.fao.org/.

[B35] Flowers T. J. (2004). Improving crop salt tolerance. *J. Exp. Bot.* 55 307–319. 10.1093/jxb/erh003 14718494

[B36] Flowers T. J. Flowers S. A. Hajibagheri M. A. Yeo A. R. (1990). Salt tolerance in halophytic wild rice, *Porteresia coarctata* Tateoka. *New Phytol.* 114 675–684. 10.1111/j.1469-8137.1990.tb00439.x

[B37] Fu C. Hu J. Liu T. Ago T. Sadoshima J. Li H. (2008). Quantitative analysis of redox-sensitive proteome with DIGE and ICAT. *J. Proteome Res.* 7 3789–3802. 10.1021/pr800233r 18707151 PMC2577071

[B38] Fukuda A. Nakamura A. Tagiri A. Tanaka H. Miyao A. Hirochika H. (2004). Function, intracellular localization and the importance in salt tolerance of a vacuolar Na^+^/H^+^ antiporter from rice. *Plant Cell Physiol.* 45 146–159. 10.1093/pcp/pch014 14988485

[B39] Fukuda M. Islam N. Woo S.-H. Yamagishi A. Takaoka M. Hirano H. (2003). Assessing matrix assisted laser desorption/ ionization-time of flight-mass spectrometry as a means of rapid embryo protein identification in rice. *Electrophoresis* 24 1319–1329. 10.1002/elps.200390168 12707926

[B40] Fumagalli E. Baldoni E. Abbruscato P. Piffanelli P. Genga A. Lamanna R. (2009). NMR techniques coupled with multivariate statistical analysis: tools to analyse *Oryza sativa* metabolic content under stress conditions. *J. Agronomy Crop Sci.* 195 77–88. 10.1111/j.1439-037X.2008.00344.x

[B41] Gagneul D. Ainouche A. Duhaze C. Lugan R. Larher F. R. Bouchereau A. (2007). A reassessment of the function of the so-called compatible solutes in the halophytic Plumbaginaceae *Limonium latifolium*. *Plant Physiol.* 144 1598–1611. 10.1104/pp.107.099820 17468212 PMC1914112

[B42] Garciadeblás B. Senn M. Banuelos M. Rodriguez-Navarro A. (2003). Sodium transport and HKT transporters: the rice model. *Plant J.* 34 788–801. 10.1046/j.1365-313X.2003.01764.x 12795699

[B43] Garg A. K. Kim J. K. Owens T. G. Ranwala A. P. Choi Y. D. Kochian L. V. (2002). Trehalose accumulation in rice plants confers high tolerance levels to different abiotic stresses. *Proc. Natl. Acad. Sci. U.S.A.* 99 15898–15903. 10.1073/pnas.252637799 12456878 PMC138536

[B44] Garg B. Puranik S. Misra S. Tripathi B. N. Prasad M. (2013). Transcript profiling identifies novel transcripts with unknown functions as primary response components to osmotic stress in wheat (*Triticum aestivum* L.) *Plant Cell Tiss. Org.* 113 91–101.

[B45] Ge H. Walhout A. J. M. Vidal M. (2003). Integrating “omic” information: a bridge between genomics and systems biology. *Trends Genet.* 19 551–560. 10.1016/j.tig.2003.08.009 14550629

[B46] Gong Q. Li P. Ma S. Rupassara S. I. Bohnert H. J. (2005). Salinity stress adaptation competence in the extremophile *Thellungiella halophila* in comparison with its relative *Arabidopsis thaliana*. *Plant J.* 44 826–839. 10.1111/j.1365-313X.2005.02587.x 16297073

[B47] Gracey A. Y. Cossins A. R. (2003). Application of microarray technology in environmental and comparative physiology. *Annu. Rev. Physiol.* 65 231–259. 10.1146/annurev.physiol.65.092101.142716 12471169

[B48] Gregorio G. B. Senadhira D. Mendoza R. D. Manigbas N. L. Roxas J. P. Guerta C. Q. (2002). Progress in breeding for salinity tolerance and associated abiotic stresses in rice. *Field Crops Res.* 76 91–101. 10.1016/S0378-4290(02)00031-X

[B49] Griffin T. J. Sherman J. Aebersold R. (2001). *Quantitative Proteomics (ICAT).* Hoboken, NJ: John Wiley & Sons Ltd.

[B50] Guo Y. Qiu Q. Quintero F. J. Pardo J. M. Ohta M. Zhang C. (2004). Transgenic evaluation of activated mutant alleles of SOS2 reveals a critical requirement for its kinase activity and C-terminal regulatory domain for salt tolerance in *Arabidopsis thaliana*. *Plant Cell* 16 435–449. 10.1105/tpc.019174 14742879 PMC341915

[B51] Hairmansis A. Berger B. Tester M. Roy A. J. (2014). Image-based phenotyping for non-destructive screening of different salinity tolerance traits in rice. *Rice* 7 16 10.1186/s12284-014-0016-3 PMC4884049 26055997

[B52] Hakim M. A. Juraimi A. S. Hanafi M. M. (2014). The effect of salinity on growth, ion accumulation anr yield of rice varieties. *J. Anim. Plant Sci.* 24 874–885.

[B53] Halfter U. Ishitani M. Zhu J. K. (2000). The *Arabidopsis* SOS2 protein kinase physically interacts with and is activated by the calcium-binding protein SOS3. *Proc. Natl. Acad. Sci. U.S.A.* 97 3735–3740. 10.1073/pnas.97.7.3735 10725350 PMC16309

[B54] Harushima Y. Yano M. Shomura A. Sato M. Shimano T. Kuboki Y. (1998). A high-density rice genetic linkage map with 2275 markers using a single F2 population. *Genetics* 148 479–494. 9475757 10.1093/genetics/148.1.479 PMC1459786

[B55] Hayashi Y. Takehisa H. Kazama Y. (2008). Characterization of salt tolerant mutants of rice induced by heavy ion irradiation. *RIKEN Accelerator Progr. Rep.* 41 234.

[B56] Hayashi Y. Takehisa H. Kazama Y. Ichida H. Ryuto H. Fukunishi N. (2007). “Effects of ion beam irradiation on mutation induction in rice,” in *Proceeding of the Eighteenth International Conference Cyclotrons and their Applications* Giardini Naxos 237–239.

[B57] Hirai M. Y. Yano M. Goodenowe D. B. Kanaya S. Kimura T. Awazuhara M. (2004). Integration of transcriptomics and metabolomics for understanding of global responses to nutritional stresses in *Arabidopsis thaliana*. *Proc. Natl. Acad. Sci. U.S.A.* 101 10205–10210. 10.1073/pnas.0403218101 15199185 PMC454188

[B58] Hoang T. M. Moghaddam L. Williams B. Khanna H. Dale J. Mundree S. G. (2015). Development of salinity tolerance in rice by constitutive-overexpression of genes involved in the regulation of programmed cell death. *Front. Plant Sci.* 6:175 10.3389/fpls.2015.00175 PMC4378369 25870602

[B59] Hollywood K. Brison D. R. Goodacre R. (2006). Metabolomics: current technologies and future trends. *Proteomics* 6 4716–4723. 10.1002/pmic.200600106 16888765

[B60] Horie T. Karahara I. Katsuhara M. (2012). Salinity tolerance mechanisms in glycophytes: an overview with the central focus on rice plants. *Rice* 5 11 10.1186/1939-8433-5-11 PMC5520831 27234237

[B61] Hoshida H. Tanaka Y. Hibino T. Hayashi Y. Tanaka A. Takabe T. (2000). Enhanced tolerance to salt stress in transgenic rice that overexpress chloroplast glutamine synthetase. *Plant Mol. Biol.* 43 103–111. 10.1023/A:1006408712416 10949377

[B62] Hu H. Dai M. Yao J. Xiao B. Li X. Zhang Q. (2006). Over expressing a NAM, ATAF, and CUC (NAC) transcription factor enhances drought resistance and salt tolerance in rice. *Proc. Natl. Acad. Sci. U.S.A.* 103 12987–12992. 10.1073/pnas.0604882103 16924117 PMC1559740

[B63] Husain S. Munns R. Condon A. G. (2003). Effect of Sodium exclusion trait on chlorophyll retention and growth of durum wheat in saline soil. *Aust. J. Agricul. Res.* 54 589–597. 10.1071/AR03032

[B64] Ito Y. Katsura K. Maruyama K. Taji T. Kobayashi M. Seki M. (2006). Functional analysis of rice DREB1/CBF-type transcription factors involved in cold-responsive gene expression in transgenic rice. *Plant Cell Physiol.* 47 141–153. 10.1093/pcp/pci230 16284406

[B65] Jain M. S. Suprasanna P. (2011). Induced mutations for enhancing nutrition and food production. *Gene Conserve* 40 201–215.

[B66] Jain S. M. Ahloowalia B. S. Veilleux R. E. (1998). “Somaclonal variation in crop plants,” in *Somaclonal Variation and Induced Mutations in Crop Improvement* eds Jain S. M. Brar D. S. Ahloowalia B. S. (Dordrecht: Kluwer Academic Publishers) 203–218. 10.1007/978-94-015-9125-6_11

[B67] Janaguiraman D. M. Ramadass R. Devi D. (2003). Effect of salt stress on germination and seedling growth in rice genotypes. *Madras Agricul. J.* 90 50–53.

[B68] Jang I. C. Oh S. J. Seo J. S. Choi W. B. Song S. I. Kim C. H. (2003). Expression of a bifunctional fusion of the *Escherichia coli* genes for trehalose-6-phosphate synthase and trehalose-6-phosphate phosphatase in transgenic rice plants increases trehalose accumulation and abiotic stress tolerance without stunting growth. *Plant Physiol.* 131 516–524. 10.1104/pp.007237 12586876 PMC166828

[B69] Jansen M. Pinto F. Nagel K. A. Dusschoten D. Fiorani F. Rascher U. (2014). “Non-invasive phenotyping methodologies enable the accurate characterization of growth and performance of shoots and roots,” in *Genomics of Plant Genetic Resources* eds Tuberosa R. Graner A. Frison E. (Dordecht: Springer) 173–206. 10.1007/978-94-007-7572-5_8

[B70] Jeschke W. D. (1984). “K^+^-Na^+^ exchange at cellular membranes, intracellular compartmentation of cations, and salt tolerance,” in *Salinity Tolerance in Plant. Strategies for Crop Improvement* eds Staple R. C. Toenniessen G. H. (New York: Wiley-Interscience Publication) 33–76.

[B71] Jiang S. Y. Bhalla R. Ramamoorthy R. Luan H. F. Venkatesh P. N. Cai M. (2012). Over-expression of OSRIP18 increases drought and salt tolerance in transgenic rice plants. *Transgenic Res.* 21 785–795. 10.1007/s11248-011-9568-9 22038450

[B72] Johnson H. E. Broadhurst D. Goodacre R. Smith A. R. (2003). Metabolic fingerprinting of salt-stressed tomatoes. *Phytochem* 62 919–928. 10.1016/S0031-9422(02)00722-7 12590119

[B73] Johnson M. K. Johnson E. J. MacElroy R. D. Speer H. L. Bru B. S. (1968). Effects of salts on the halophilic alga *Dunaliella viridis*. *J. Bacteriol.* 95 1461–1468. 5646631 10.1128/jb.95.4.1461-1468.1968 PMC315107

[B74] Kaplan F. Kopka J. Haskell D. W. Zhao W. Schiller K. C. Gatzke N. (2004). Exploring the temperature-stress metabolome of *Arabidopsis*. *Plant Physiol.* 136 4159–4168. 10.1104/pp.104.052142 15557093 PMC535846

[B75] Kempa S. Krasensky J. Dal Santo S. Kopka J. Jonak C. (2008). A central role of abscisic acid in stress-regulated carbohydrate metabolism. *PLoS ONE* 3:e3935 10.1371/journal.pone.0003935 PMC2593778 19081841

[B76] Khatun S. Flowers T. J. (1995). The estimation of pollen viability in rice. *J. Exp. Bot.* 46 151–154. 10.1093/jxb/46.1.151

[B77] Kim H. K. Choi Y. H. Verpoorte R. (2010). NMR-based metabolomic analysis of plants. *Nat. Protoc. U.S.A.* 5 536–549. 10.1038/nprot.2009.237 20203669

[B78] Koller A. Washburn M. P. Lange B. M. Andon N. L. Deciu C. Haynes P. A. (2002). Proteomic survey of metabolic pathways in rice. *Proc. Natl. Acad. Sci. U.S.A.* 99 11969–11974. 10.1073/pnas.172183199 12163647 PMC129378

[B79] Komatsu S. Yamamoto R. Nanjo Y. Mikami Y. Yunokawa H. Sakata K. (2009). A comprehensive analysis of the soybean genes and proteins expressed under flooding stress using transcriptome and proteome techniques. *J. Proteom. Res.* 8 4766–4778. 10.1021/pr900460x 19658438

[B80] Koyama M. L. Levesley A. Koebner R. M. D. Flowers T. J. Yeo A. R. (2001). Quantitative trait loci for component physiological traits determining salt tolerance in rice. *Plant Physiol.* 125 406–422. 10.1104/pp.125.1.406 11154348 PMC61021

[B81] Krishnamurthy P. Ranathunge K. Franke R. Prakash H. S. Schreiber L. Mathew M. K. (2009). The role of root apoplastic transport barriers in salt tolerance of rice (*Oryza sativa* L.). *Planta* 230 119–134. 10.1007/s00425-009-0930-6 19363620

[B82] Kumar G. Kushwaha H. R. Sabharwal V. P. Kumari S. Joshi R. Karan R. (2012). Clustered metallothionein genes are co-regulated in rice and ectopic expression of OsMT1e-P confers multiple abiotic stress tolerance in tobacco via ROS scavenging. *BMC Plant Biol.* 12:107 10.1186/1471-2229-12-107 PMC3491035 22780875

[B83] Kumar K. Kumar M. Kim S.-R. Ryu H. Cho Y.-G. (2013). Insights into genomics of salt stress response in rice. *Rice* 6 27–41. 10.1186/1939-8433-6-27 24280112 PMC4883734

[B84] Kumari S. Roy S. Singh P. Singla-Pareek S. L. Pareek A. (2013). Cyclophilins: proteins in search of function. *Plant Sign. Behav.* 8 8–15. 10.4161/psb.22734 PMC3745578 23123451

[B85] Kumari S. Sabharwal V. P. Khushwaha H. R. Sopory S. K. Singla-Pareek S. L. Pareek A. (2009). Transcriptome map for seedling stage specific salinity stress response indicates a specific set of genes as candidate for saline tolerance in *Oryza sativa* L. *Funct. Integr. Genomics.* 9 109–123. 10.1007/s10142-008-0088-5 18594887

[B86] Kurata N. Nakamura Y. Yamamoto K. Harushima Y. Sue N. Wu J. (1994). A 300 kilobase interval genetic map of rice including 883 expressed sequences. *Nat. Genet.* 8 365–372. 10.1038/ng1294-365 7894488

[B87] Kusano M. Fukushima A. Kobayashi M. Hayashi N. Jonsson P. Moritz T. (2007). Application of a metabolomic method combining one-dimensional and two-dimensional gas chromatography-time-of-flight/mass spectrometry to metabolic phenotyping of natural variants in rice. *J. Chromatogr. B Biomed. Sci. Appl.* 855 71–79. 10.1016/j.jchromb.2007.05.002 17556050

[B88] Lacan D. Durand M. (1996). Na^+^-K^+^ exchange at the xylem/symplast boundary (its significance in the salt sensitivity of soybean). *Plant Physiol.* 110 705–711. 12226212 10.1104/pp.110.2.705 PMC157767

[B89] Lee D. G. Ahsan N. Lee S. H. Lee J. J. Bahk J. D. Kang K. Y. (2009). Chilling stress-induced proteomic changes in rice roots. *J. Plant Physiol.* 166 1–11. 10.1016/j.jplph.2008.02.001 18433929

[B90] Leung H. (2008). Stressed genomics-bringing relief to rice fields. *Curr. Opin. Plant Biol.* 11 201–208. 10.1016/j.pbi.2007.12.005 18294900

[B91] Lin H. X. Zhu M. Z. Yano M. Gao J. P. Liang Z. W. Su W. A. (2004). QTLs for Na^+^ and K^+^ uptake of the shoots and roots controlling rice salt tolerance. *Theor. Appl. Genet.* 108 253–260. 10.1007/s00122-003-1421-y 14513218

[B92] Liu A. L. Zou J. Liu C. F. Zhou X. Y. Zhang X. W. Luo G. Y. (2013). Over-expression of OsHsfA7 enhanced salt and drought tolerance in transgenic rice. *BMB Rep.* 46 31–36. 10.5483/BMBRep.2013.46.1.090 23351381 PMC4133825

[B93] Liu G. Li X. Jin S. Liu X. Zhu L. Nie Y. (2014a). Overexpression of rice NAC gene SNAC1 improves drought and salt tolerance by enhancing root development and reducing transpiration rate in transgenic cotton. *PLoS ONE* 9:e86895 10.1371/journal.pone.0086895 PMC3904958 24489802

[B94] Liu C. Mao B. Ou S. Wang W. Liu L. Wu Y. (2014b). OsbZIP71, a bZIP transcription factor, confers salinity and drought tolerance in rice. *Plant Mol. Biol.* 84 19–36. 10.1007/s11103-013-0115-3 23918260

[B95] Liu J. Zhu J. K. (1998). A calcium sensor homolog required for plant salt tolerance. *Science* 280 1943–1945. 10.1126/science.280.5371.1943 9632394

[B96] Liu J. X. Bennett J. (2011). Reversible and irreversible drought-induced changes in the anther proteome of rice (*Oryza sativa* L.) genotypes IR64 and moroberekan. *Mol. Plant* 4 59–69. 10.1093/mp/ssq039 20643753

[B97] Lugan R. Niogret M. F. Leport L. Guegan J. P. Larher F. R. Savoure A. (2010). Metabolome and water homeostasis analysis of *Thellungiella salsuginea* suggests that dehydration tolerance is a key response to osmotic stress in this halophyte. *Plant J.* 64 215–229. 10.1111/j.1365-313X.2010.04323.x 21070405

[B98] Lutts S. Kinet J. M. Bouharmont J. (1995). Changes in plant response to NaCl during development of rice (*Oryza sativa* L.) Varieties differing in salinity resistance. *J. Exp. Bot.* 46 1843–1852. 10.1093/jxb/46.12.1843

[B99] Ma L. Zhang H. Sun L. Jiao Y. Zhang G. Miao C. (2012). NADPH oxidase AtrbohD and AtrbohF function in ROS-dependent regulation of Na^+^/K^+^ homeostasis in *Arabidopsis* under salt stress. *J. Exp. Bot.* 63 305–317. 10.1093/jxb/err280 21984648

[B100] Ma N. L. Rahmat Z. Lam S. S. (2013). A review of the “Omics” approach to biomarkers of oxidative stress in *Oryza sativa*. *Int. J. Mol. Sci.* 14 7515–7541. 10.3390/ijms14047515 23567269 PMC3645701

[B101] Ma X. Qian Q. Zhu D. (2005). Expression of a calcineurin gene improves salt stress tolerance in transgenic rice. *Plant Mol. Biol.* 58 483–495. 10.1007/s11103-005-6162-7 16021334

[B102] Maas E. V. Hoffman G. J. (1977). Crop salt tolerance, current assessment. *J. Irrig. Drain. E-ASCE.* 103 115–134.

[B103] Mahmod A. Latif T. Khan M. A. (2009). Effect of salinity on growth, yield and yield components in basmati rice germplasm. *Pak. J. Bot.* 41 3035–3045.

[B104] Makela P. Karkkainen J. Somersalo S. (2000). Effect of glycine betaine on chloroplast ultrastructure, chlorophyll and protein content, and RuBPCO activities in tomato grown under drought or salinity. *Biol. Plant.* 43 471–475. 10.1023/A:1026712426180

[B105] Malik V. Wu R. (2005). Transcription factor AtMyb2 increased salt-stress tolerance in rice (*Oryza sativa* L.). *Rice Genet. Newslett.* 22 63.

[B106] Mantri N. Patade V. Penna S. Ford R. Pang E. (2012). “Abiotic stress responses in plants: present and future,” in *Abiotic Stress Responses in Plants: Metabolism, Productivity and Sustainability* eds Ahmad P. Prasad M. N. V. (New York: Springer) 1–19.

[B107] Matsumoto T. Wu J. Z. Kanamori H. Katayose Y. Fujisawa M. Namiki N. (2005). The map-based sequence of the rice genome. *Nature* 436 793–800. 10.1038/nature03895 16100779

[B108] McCouch S. R. Kochert G. Yu Z. H. Wang Z. Y. Khush G. S. Coffman W. R. (1988). Molecular mapping of rice chromosomes. *Theor. Appl. Genet.* 76 815–829. 10.1007/BF00273666 24232389

[B109] Mochida K. Shinozaki K. (2011). Advances in omics and bioinformatics tools for systems analyses of plant functions. *Plant Cell Physiol.* 52 2017–2038. 10.1093/pcp/pcr153 22156726 PMC3233218

[B110] Mohammadi-Nejad G. Singh R. K. Arzanic A. Rezaiec A. M. Sabourid H. Gregorio G. B. (2010). Evaluation of salinity tolerance in rice genotypes. *Int. J. Plant Prod.* 4 199–207.

[B111] Mohan M. Narayanan M. S. L. Ibrahim S. M. (2000). Chlorophyll stability index (CSI): its impact on salt tolerance in rice. *Int. Rice Res. Notes* 25 38–39.

[B112] Mohanty A. Kathuria H. Ferjani A. Sakamoto A. Mohanty P. Murata N. (2002). Transgenics of an elite indica rice variety Pusa Basmati 1 harbouring the codA gene are highly tolerant to salt stress. *Theor. Appl. Genet.* 106 51–57. 12582870 10.1007/s00122-002-1063-5

[B113] Motohashi T. Nagamiya K. Prodhan S. H. Nakao K. Shishido T. Yamamoto Y. (2010). Production of salt stress tolerant rice by overexpression of the catalase gene, katE, derived from *Escherichia coli*. *J. Mol. Biol. Biotechnol.* 18 37–41.

[B114] Munns R. (1985). Na^+^, K^+^ and Cl^-^ in xylem sap flowing to shoots of NaCl-treated barley. *J. Exp. Bot.* 36 1032–1042. 10.1093/jxb/36.7.1032

[B115] Munns R. James R. A. (2003). Screening methods for salinity tolerance: a case study with tetraploid wheat. *Plant Soil.* 253 201–218. 10.1023/A:1024553303144

[B116] Munns R. James R. A. Sirault X. R. R. Furbank R. T. Jones H. G. (2010). New phenotyping methods for screening wheat and barley for beneficial responses to water deficit. *J. Exp. Bot.* 61 3499–3507. 10.1093/jxb/erq199 20605897

[B117] Munns R. Tester M. (2008). Mechanisms of salinity tolerance. *Annu. Rev. Plant Biol.* 59 651–681. 10.1146/annurev.arplant.59.032607.092911 18444910

[B118] Nagamiya K. Motohashi T. Nakao K. Prodhan S. H. Hattori E. Hirose S. (2007). Enhancement of salt tolerance in transgenic rice expressing an *Escherichia coli* catalase gene, katE. *Plant Biotech. Rep.* 1 49–55. 10.1007/s11816-007-0007-6

[B119] Nejad G. M. Singh R. K. Arzani Rezaie A. A. M. Sabourid H. Gregorio G. B. (2010). Evaluation of salinity tolerance in rice genotypes. *Int. J. Plant Prod.* 4 1735–1743.

[B120] Novak H. J. Brunner H. (1992). Plant breeding: Induced mutation technology for crop improvement. *IAEA Bull.* 4 25–33.

[B121] Obata T. Kitamoto H. K. Nakamura A. Fukuda A. Tanaka Y. (2007). Rice shaker potassium channel OsKAT1 confers tolerance to salinity stress on yeast and rice cells. *Plant Physiol.* 144 1978–1985. 10.1104/pp.107.101154 17586689 PMC1949902

[B122] Ohta M. Hayashia Y. Nakashimaa A. Hamada A. Tanaka A. Nakamurab T. (2002). Introduction of a Na^+^/H^+^ antiporter gene from *Atriplex gmelini* confers salt tolerance to rice. *FEBS Lett.* 532 279–282. 10.1016/S0014-5793(02)03679-7 12482579

[B123] Okazaki Y. Saito K. (2012). Recent advances of metabolomics in plant biotechnology. *Plant Biotechnol. Rep.* 6 1–15. 10.1007/s11816-011-0191-2 22308170 PMC3262138

[B124] Pareek A. Sopory S. K. Bohnert H. J. Govindjee (eds). (2010). *Abiotic Stress Adaptation in Plants: Physiological, Molecular and Genomic Foundation.* Berlin: Springer 10.1007/978-90-481-3112-9

[B125] Parry M. A. J. Madgwick P. J. Bayon C. Tearall K. Lopez A. H. Baudo M. (2009). Mutation discovery for crop improvement. *J. Exp. Bot.* 60 2817–2825. 10.1093/jxb/erp189 19516074

[B126] Peng X. Ding X. Chang T. Wang Z. Liu R. Zeng X. (2014). Overexpression of a vesicle trafficking gene, OsRab7, enhances salt tolerance in rice. *Sci. World J.* 2014 483526 10.1155/2014/483526 PMC3943248 24688390

[B127] Plett J. M. Kemppainen M. Kale S. D. Kohler A. Legue V. Brun A. (2011). A secreted effector protein of *Laccaria bicolor* is required for symbiosis development. *Curr. Biol.* 21 1197–1203. 10.1016/j.cub.2011.05.033 21757352

[B128] Prasad S. R. Bagali P. G. Hittalmani S. Shashidhar H. E. (2000). Molecular mapping of quantitative trait loci associated with seedling tolerance to salt stress in rice (*Oryza sativa* L.). *Curr. Sci.* 78 162–164.

[B129] Prashanth S. R. Sadhasivam V. Parida A. (2008). Overexpression of cytosolic copper/zinc superoxide dismutase from a mangrove plant *Avicennia marina* in indica Rice var Pusa Basmati-1 confers abiotic stress tolerance. *Transgenic Res.* 17 281–291. 10.1007/s11248-007-9099-6 17541718

[B130] Quintero F. J. Ohta M. Shi H. Zhu J.-K. Pardo J. M. (2002). Reconstitution in yeast of the *Arabidopsis* SOS signaling pathway for Na^+^ homeostasis. *Proc. Natl. Acad. Sci. U.S.A.* 99 9061–9066. 10.1073/pnas.132092099 12070350 PMC124423

[B131] Qureshi R. H. Barrett-Lennard E. G. (1998). “Crops and grasses for salt affected land,” in *Saline Agriculture for Irrigated Land in Pakistan: A Handbook* eds Qureshi R. H. Barrett-Lennard E. G. (Canberra: ACIAR Monograph) 51–62.

[B132] Rachmat G. Nugroho S. Sukma D. Aswidinnoor H. Sudarsono S. (2014). Overexpression of OsNAC6 transcription factor from Indonesia rice cultivar enhances drought and salt tolerance. *Emir. J. Food Agric.* 26 519–527. 10.9755/ejfa.v26i6.17672

[B133] Rajendran K. Tester M. Roy S. J. (2009). Quantifying the three main components of salinity tolerance in cereals. *Plant Cell Environ.* 32 237–249. 10.1111/j.1365-3040.2008.01916.x 19054352

[B134] Ranathunge K. Kotula L. Steudle E. Lafitte R. (2004). Water permeability and reflection coefficient of the outer part of young rice roots are differently affected by closure of water channels (aquaporins) or blockage of apoplastic pores. *J. Exp. Bot.* 55 433–447. 10.1093/jxb/erh041 14739266

[B135] Ranathunge K. Steudle E. Lafitte R. (2003). Control of water uptake by rice (*Oryza sativa* L.): role of the outer part of the root. *Planta* 217 193–205. 12783327 10.1007/s00425-003-0984-9

[B136] Ranathunge K. Steudle E. Lafitte R. (2005). Blockage of apoplastic bypass-flow of water in rice roots by insoluble salt precipitates analogous to a Pfeffer cell. *Plant Cell Environ.* 28 121–133. 10.1111/j.1365-3040.2004.01245.x

[B137] Ray S. Satya P. (2014). Next generation sequencing technologies for next generation plant breeding. *Front. Plant Sci.* 5:367 10.3389/fpls.2014.00367 PMC4115663 25126091

[B138] Ren Z. H. Gao J. P. Li L. G. Cai X. L. Huang W. Chao D. Y. (2005). A rice quantitative trait locus for salt tolerance encodes a sodium transporter. *Nat. Genet.* 37 1141–1146. 10.1038/ng1643 16155566

[B139] Reynolds M. P. Ortiz-Monasterio J. I. Mc Nab A. (2001). *Application of Physiology in Wheat Breeding.* Mexico, DF: CIMMYT.

[B140] Rohila J. S. Jain R. K. Wu R. (2002). Genetic improvement of basmati rice for salt and drought tolerance by regulated expression of a barley HVA1 cDNA. *Plant Sci.* 163 525–532. 10.1016/S0168-9452(02)00155-3

[B141] Roy M. Wu R. (2001). Arginine decarboxylase transgene expression and analysis of environmental stress tolerance in transgenic rice. *Plant Sci.* 160 869–875. 10.1016/S0168-9452(01)00337-5 11297783

[B142] Royo A. Abio D. (2003). Salt tolerance in durum wheat cultivars. *Span. J. Agric. Res.* 1 27–35. 10.5424/sjar/2003013-32

[B143] Royuela M. Gonzalez A. Gonzalez E. M. Arrese-Igor C. Aparicio-Tejo P. M. Gonzalez-Murua C. (2000). Physiological consequences of continuous, sublethal imazethapyr supply to pea plants. *J. Plant Physiol.* 157 345–354. 10.1016/S0176-1617(00)80057-7

[B144] Sahi C. Singh A. Blumwald E. Grover A. (2006). Beyond osmolytes and transporters: novel plant salt stress tolerance-related genes from transcriptional profiling data. *Physiol. Plant.* 127 1–9. 10.1111/j.1399-3054.2005.00610.x

[B145] Saijo Y. Hata S. Kyozuka J. Shimamoto K. Izui K. (2000). Overexpression of a single Ca2+ dependent protein kinase confers both cold and salt/drought tolerance on rice plants. *Plant J.* 23 319–327. 10.1046/j.1365-313x.2000.00787.x 10929125

[B146] Saleem M. Y. Mukhtar Z. Cheema A. A. Atta B. M. (2005). Induced mutation and in vitro techniques as a method to induce salt tolerance in Basmati rice (*Oryza sativa* L.). *Int. J. Environ. Sci. Tech.* 2 141–145. 10.1007/BF03325868

[B147] Sanders D. (2000). Plant biology: the salty tale of *Arabidopsis*. *Curr. Biol.* 10 R486–R488. 10.1016/S0960-9822(00)00554-6 10898972

[B148] Schreiber L. Franke R. Hartmann K. D. Ranathunge K. Steudle E. (2005). The chemical composition of suberin in apoplastic barriers affects radial hydraulic conductivity differently in the roots of rice (*Oryza sativa* L. *cv.* IR64) and corn (*Zea mays* L. cv. Helix). *J. Exp. Bot.* 56 1427–1436. 10.1093/jxb/eri144 15809280

[B149] Schreiber L. Hartmann K. Skrabs M. Zeier Jr. (1999). Apoplastic barriers in roots: chemical composition of endodermal and hypodermal cell walls. *J. Exp. Bot.* 50 1267–1280. 10.1093/jxb/50.337.1267

[B150] Schroeder J. I. Delhaize E. Frommer W. B. Guerinot M. L. Harrison M. J. Harrera-Estrella L. (2013). Using membrane transporters to improve crops for sustainable food production. *Nature* 497 60–66. 10.1038/nature11909 23636397 PMC3954111

[B151] Senguttuvel P. Vijayalakshmi C. Thiyagarajan K. Kannanbapu J. R. Kota S. Padmavathi G. (2014). Changes in photosynthesis, chlorophyll fluorescence, gas exchange parameters and osmotic potential to salt stress during early seedling stage in rice (*Oryza sativa* L.). *SABRAO J. Breed. Genet.* 46 120–135.

[B152] Shannon M. C. Rhoades J. D. Draper J. H. Scardaci S. C. Spyres M. D. (1998). Assessment of salt tolerance in rice cultivars in response to salinity problems in California. *Crop Sci.* 38 394–398. 10.2135/cropsci1998.0011183X003800020021x

[B153] Shu X. L. Frank T. Shu Q. Y. Engel K. H. (2008). Metabolite profiling of germinating rice seeds *J. Agric. Food Chem.* 56 11612–11620. 10.1021/jf802671p 19053355

[B154] Sikuku P. A. Netondo G. W. Onyango J. C. Musyimi D. M. (2010). Chlorophyll fluorescence, protein and chlorophyll content of three NERICA rainfed rice varieties under varying irrigation regimes. *ARPN J. Agr. Biol. Sci.* 5 19–25.

[B155] Singh A. K. Ansari M. W. Pareek A. Singla-Pareek S. L. (2008). Raising salinity tolerant rice: recent progress and future perspectives. *Physiol. Mol. Biol. Plants.* 14 137–154. 10.1007/s12298-008-0013-3 23572881 PMC3550660

[B156] Singh B. D. (2000). *Plant Breeding-Principles and Methods.* New Delhi: Kalyani Publisher, Narosa Publishing House.

[B157] Singh R. K. Mishra B. Singh K. N. (2004). Salt tolerant rice varieties and their role in reclamation programme in Uttar Pradesh. *Indian Far.* 6–10.

[B158] Singla-Pareek S. L. Yadav S. K. Pareek A. Reddy M. K. Sopory S. K. (2008). Enhancing salt tolerance in a crop plant by overexpression of glyoxalase II. *Transgenic Res.* 17 171–180. 10.1007/s11248-007-9082-2 17387627

[B159] Sirault X. R. R. James R. A. Furbank R. T. (2009). A new screening method for osmotic component of salinity tolerance in cereals using infrared thermography. *Funct. Plant Biol.* 36 970–977. 10.1071/FP09182 32688708

[B160] Soda N. Kushwaha H. R. Soni P. Singla–Pareek S. L. Pareek A. (2013). A suite of new genes defining salinity stress tolerance in seedlings of contrasting rice genotypes. *Funct. Integr. Genomics.* 13 351–365. 10.1007/s10142-013-0328-1 23813016

[B161] Staal M. Maathuis F. J. M. Elzenga J. T. M. Overbeek J. H. M. Prins H. B. A. (1991). Na^+^/H^+^ antiport activity in tonoplast vesicles from roots of the salt-tolerant plantago maritima and the salt-Sensitive plantago media. *Physiol. Plant.* 82 179–184. 10.1111/j.1399-3054.1991.tb00078.x

[B162] Su J. Hirji R. Zhang L. He C. Selvaraj G. Wu R. (2006). Evaluation of the stress-inducible production of choline oxidase in transgenic rice as a strategy for producing the stress-protectant glycine betaine. *J. Exp. Bot.* 57 1129–1135. 10.1093/jxb/erj133 16510513

[B163] Su J. Wu R. (2004). Stress-inducible synthesis of proline in transgenic rice confers faster growth under stress conditions than that with constitutive synthesis. *Plant Sci.* 166 941–948. 10.1016/j.plantsci.2003.12.004

[B164] Tarpley L. Duran A. Kebrom T. Sumner L. (2005). Biomarker metabolites capturing the metabolite variance present in a rice plant developmental period. *BMC Plant Biol* 5:8 10.1186/1471-2229-5-8 PMC1175851 15927065

[B165] Thomas J. C. Sepahi M. Arendall B. Bohnert H. J. (1995). Enhancement of seed germination in high salinity by engineering mannitol expression in *Arabidopsis thaliana*. *Plant Cell Environ.* 18 801–806. 10.1111/j.1365-3040.1995.tb00584.x

[B166] Thompson G. A. Goggin F. L. (2006). Transcriptomics and functional genomics of plant defence induction by phloem-feeding insects. *J. Exp. Bot.* 57 755–766. 10.1093/jxb/erj135 16495409

[B167] Todaka D. Nakashima K. Shinozaki K. Yamaguchi-Shinozaki K. (2012). Towards understanding transcriptional regulatory networks in abiotic stress responses and tolerance in rice. *Rice* 5 6 10.1186/1939-8433-5-6 PMC3834508 24764506

[B168] Tuncbag N. McCallum S. Huang S. S. C. Fraenkel E. (2012). SteinerNet: a web server for integrating “omic” data to discover hidden components of response pathways. *Nucleic Acids Res.* 40 W505–W509. 10.1093/nar/gks445 22638579 PMC3394335

[B169] Urano K. Maruyama K. Ogata Y. Morishita Y. Takeda M. Sakurai N. (2009). Characterization of the ABA-regulated global responses to dehydration in *Arabidopsis* by metabolomics. *Plant J.* 57 1065–1078. 10.1111/j.1365-313X.2008.03748.x 19036030

[B170] Usadel B. Blasing O. E. Gibon Y. Poree F. Hohne M. Gunter M. (2008). Multilevel genomic analysis of the response of transcripts, enzyme activities and metabolites in *Arabidopsis* rosettes to a progressive decrease of temperature in the non-freezing range. *Plant Cell Environ.* 31 518–547. 10.1111/j.1365-3040.2007.01763.x 18088337

[B171] USDA-ARS. (2005). *National Fluoride Database of Selected Beverages and Foods - Release 2* (2005). Available at: http://www.ars.usda.gov/Services/docs.htm

[B172] Verma D. Singla-Pareek S. L. Rajagopal D. Reddy M. K. Sopory S. K. (2007). Functional validation of a novel isoform of Na^+^/H^+^ antiporter from *Pennisetum glaucum* for enhancing salinity tolerance in rice. *J. Biosci.* 32 621–628. 10.1007/s12038-007-0061-9 17536181

[B173] Wakasa K. Hasegawa H. Nemoto H. Matsuda F. Miyazawa H. Tozawa Y. (2006). High-level tryptophan accumulation in seeds of transgenic rice and its limited effects on agronomic traits and seed metabolite profile. *J. Exp. Bot.* 57 3069–3078. 10.1093/jxb/erl068 16908506

[B174] Walia H. Wilson C. Zeng L. Ismail A. M. Condamine P. Close T. J. (2007). Genome-wide transcriptional analysis of salinity stressed japonica and indica rice genotypes during panicle initiation stage. *Plant Mol. Biol.* 63 609–623. 10.1007/s11103-006-9112-0 17160619 PMC1805040

[B175] Wang W. X. Vinocur B. Altman A. (2003). Plant responses to drought, salinity and extreme temperatures: towards genetic engineering for stress tolerance. *Planta* 218 1–14. 10.1007/s00425-003-1105-5 14513379

[B176] Wilson P. Wolfe A. D. Armbruster W. S. Thomson J. D. (2007). Constrained lability in floral evolution: counting convergent origins of hummingbird pollination in *Penstemon* and *Keckiella*. *New Phytol.* 176 883–890. 10.1111/j.1469-8137.2007.02219.x 17897322

[B177] Xiong L. Yang Y. (2003). Disease resistance and abiotic stress tolerance in rice are inversely modulated by an abscisic acid–inducible mitogen-activated protein kinase. *Plant Cell* 15 745–759. 10.1105/tpc.008714 12615946 PMC150027

[B178] Xiong L. Zhu J. K. (2002). Molecular and genetic aspects of plant responses to osmotic stress. *Plant Cell Environ.* 25 131–139. 10.1046/j.1365-3040.2002.00782.x 11841658

[B179] Xu D. Duan X. Wang B. Hong B. Ho T. H. D. Wu R. (1996). Expression of a late embryogenesis abundant (LEA) protein gene, HVA1, from barley confers tolerance to drought and salinity in transgenic rice. *Plant Physiol.* 110 249–257. 12226181 10.1104/pp.110.1.249 PMC157716

[B180] Xu G. Cui Y. Li M. Wang M. Yu Y. Zhang B. (2013). OsMSR2, a novel rice calmodulin-like gene, confers enhanced salt tolerance in rice (*Oryza sativa* L.). *Aust. J. Crop Sci.* 7 368–373.

[B181] Yeo A. R. (1999). Predicting the interaction between the effects of salinity and climate change on crop plants. *Sci. Hortic.* 78 159–174. 10.1016/S0304-4238(98)00193-9

[B182] Yeo A. R. Caporn S. J. M. Flowers T. J. (1985). The effect of salinity upon photosynthesis in rice (*Oryza sativa* L.)*:* gas exchange by individual leaves in relation to their salt content. *J. Exp. Bot.* 36 1240–1248. 10.1093/jxb/36.8.1240

[B183] Yeo A. R. Yeo M. E. Flowers S. A. Flowers T. J. (1990). Screening of rice (*Oryza sativa* L.) genotypes for physiological characters contributing to salinity resistance, and their relationship to overall performance. *Theor. Appl. Genet.* 79 377–384. 10.1007/BF01186082 24226357

[B184] Yeo A. R. Yeo M. E. Flowers T. J. (1987). The contribution of an apoplastic pathway to sodium uptake by rice roots in saline conditions. *J. Exp. Bot.* 38 1141–1153. 10.1093/jxb/38.7.1141

[B185] Zeng L. Poss J. Wilson C. Draz A.-S. Gregorio G. Grieve C. (2003). Evaluation of salt tolerance in rice genotypes by physiological characters. *Euphytica.* 129 281–292. 10.1023/A:1022248522536

[B186] Zhang H. X. Hodson J. N. Williams J. P. Blumwald E. (2001). Engineering salt-tolerant *Brassica* plants: characterization of yield and seed oil quality in transgenic plants with increased vacuolar sodium accumulation. *Proc. Natl. Acad. Sci. U.S.A.* 98 12832–12836. 10.1073/pnas.231476498 11606781 PMC60139

[B187] Zhang Q. Gao Y. J. Saghai Maroof M. A. Yang S. H. Li J. X. (1995). Molecular divergence and hybrid performance in rice. *Mol. Breed.* 1 133–142. 10.1007/BF01249698

[B188] Zhao F. Y. Guo S. L. Zhang H. Zhao Y. X. (2006a). Expression of yeast SOD2 in transgenic rice results in increased salt tolerance. *Plant Sci.* 170 216–224. 10.1016/j.plantsci.2005.08.017

[B189] Zhao F. Wang Z. Zhang Q. Zhao Y. Zhang H. (2006b). Analysis of the physiological mechanism of salt-tolerant transgenic rice carrying a vacuolar Na^+^/H^+^ antiporter gene from *Suaeda salsa*. *J. Plant Res.* 119 95–104. 10.1007/s10265-005-0250-2 16565882

[B190] Zhao F. Zhang H. (2006). Salt and paraquat stress tolerance results from co-expression of the *Suaeda salsa* glutathione S-transferase and catalase in transgenic rice. *Plant Cell Tissue Org. Cult.* 86 349–358. 10.1007/s11240-006-9133-z

[B191] Zhu J. K. Liu J. P. Xiong L. M. (1998). Genetic analysis of salt tolerance in *Arabidopsis*: evidence for a critical role of potassium nutrition. *Plant Cell* 10 1181–1191. 10.1105/tpc.10.7.1181 9668136 PMC144057

[B192] Zuther E. Koehl K. Kopka J. (2007). “Comparative metabolome analysis of the salt response in breeding cultivars of rice,” in *Advances in Molecular Breeding Toward Drought and Salt Tolerant Crops* eds Jenk M. A. Hasegawa P. M. Jain S. M. (Berlin: Springer) 285–315. 10.1007/978-1-4020-5578-2_12

